# Antimicrobial Resistance in Equines: A Growing Threat to Horse Health and Beyond—A Comprehensive Review

**DOI:** 10.3390/antibiotics13080713

**Published:** 2024-07-29

**Authors:** Ajran Kabir, Bibek Lamichhane, Tasmia Habib, Alexis Adams, Hossam El-Sheikh Ali, Nathan M. Slovis, Mats H. T. Troedsson, Yosra A. Helmy

**Affiliations:** 1 Maxwell H. Gluck Equine Research Center, Department of Veterinary Science, Martin-Gatton College of Agriculture, Food and Environment, University of Kentucky, Lexington, KY 40546, USA; ajran.kabir@uky.edu (A.K.);; 2 College of Veterinary Medicine, Lincoln Memorial University, Harrogate, TN 37752, USA; 3 McGee Medical Center, Hagyard Equine Medical Institute, 4250 Iron Works Pike, Lexington, KY 40511, USA; nslovis@hagyard.com

**Keywords:** horses, antimicrobial resistance, antimicrobial stewardship, epidemiology, One Health

## Abstract

The equine industry holds substantial economic importance not only in the USA but worldwide. The occurrence of various infectious bacterial diseases in horses can lead to severe health issues, economic losses, and restrictions on horse movement and trade. Effective management and control of these diseases are therefore crucial for the growth and sustainability of the equine industry. While antibiotics constitute the primary treatment strategy for any bacterial infections in horses, developing resistance to clinically important antibiotics poses significant challenges to equine health and welfare. The adverse effects of antimicrobial overuse and the escalating threat of resistance underscore the critical importance of antimicrobial stewardship within the equine industry. There is limited information on the epidemiology of antimicrobial-resistant bacterial infections in horses. In this comprehensive review, we focus on the history and types of antimicrobials used in horses and provide recommendations for combating drug-resistant bacterial infections in horses. This review also highlights the epidemiology of antimicrobial resistance (AMR) in horses, emphasizing the public health significance and transmission dynamics between horses and other animals within a One Health framework. By fostering responsible practices and innovative control measures, we can better help the equine industry combat the pressing threat of AMR and thus safeguard equine as well as public health.

## 1. Introduction

Equines form a major part of the livestock industry, playing a critical role in the economic landscape both nationally and internationally [1]. The United States (U.S.) Agriculture Improvement Act of 2018 primarily categorizes horses as “livestock”. Additionally, the legislation ensures that horses are not generally defined as “pets” within the Pet and Women Safety (PAWS) Act, though they are acknowledged similarly to service animals and emotional support animals [2]. In 2017, the American Horse Council (AHC) revealed that the equine industry alone contributed a total of approximately $122 billion to the national economy. This increased to $177 billion by 2023, highlighting the growing economic influence of the equine industry [3,4]. In the USA, approximately 7.25 million horses and 1.74 million people were employed within the equine industry [5]. The equine industry directly supports five major business sectors: agriculture and livestock, hospitality (including hotels and motels), real estate, commercial sports (horse racing), and wholesale trade [4]. According to the AHC, the commercial horse sporting sector contributed approximately $28.3 billion to the national gross domestic product (GDP), which is approximately 23.2% of the GDP generated by the horse industry in 2017 [6]. Beyond these areas, the equine industry historically plays an essential role in non-agricultural sectors, such as construction, tourism, and safari operations; public transport; goods transportation; mining; carting; carriage services; and police and military/paramilitary forces globally [7]. Horses significantly enhance safari operations by offering unique, immersive experiences and access to areas inaccessible by vehicles, allowing for closer wildlife encounters. Their use also adds authenticity and minimizes environmental impact, providing an eco-friendly and sustainable adventure [8,9]. The significant potential of the equine industry for income-generating activities is due to its versatility and wide range of applications, all of which are dependent on the maintenance of equine health and an effective strategy against the transmission of infectious diseases. However, their current role is often limited by various health concerns, and they are frequently overlooked in agricultural policy, research, and education initiatives. This exclusion has ultimately limited the economic potential and development of comprehensive strategies for the equine industry, as their management and care have been relatively slow. In fact, the presence of various infectious diseases is an important limiting factor for the overall development and expansion of the equine industry [10]. Since horses are susceptible to a wide range of infectious diseases, the majority of which are caused by various bacterial pathogens [10], the treatment of existing bacterial infections mainly relies on antibiotics. However, the effective prevention of bacterial infections depends on other factors such as biosecurity and prophylaxis using vaccines or antibiotics [11,12].

The discovery of antibiotics in 1928 revolutionized modern medicine. The antibiotics were referred to as “miracle drugs”. This discovery fundamentally transformed therapeutic practices by enabling the effective treatment of bacterial infections and significantly improving health outcomes [13]. Beyond their medical applications, antibiotics play a crucial role in the animal production sector, where they are used globally for disease prevention and as growth promoters. However, bacteria have developed resistance to these antibiotic drugs over time [14]. The incredible adaptive ability of the bacteria enables them to adjust to challenging environmental circumstances, including the presence of antibiotics. This dynamic nature allows bacteria to swiftly adapt to stressors, which is a key factor in driving the development of antibiotic resistance, survival, and persistence [15]. Antimicrobial resistance (AMR) is the capability of bacteria to grow and proliferate despite the presence of antibiotic drugs that are intended to kill them [16,17]. Bacteria develop resistance to antibiotics when they are exposed to them at a therapeutic level or at a low/sub-lethal level for a long period of time [18]. The development of AMR is mainly due to overuse and misuse of clinically essential antibiotics, as well as unnecessarily use or a lack of evidence-based diagnosis and treatment with antibiotics without a prescription [19]. According to the World Health Organization (WHO), AMR is one of the major threats to global health and is considered one of the leading causes of death in the world after climate change, pollution, and cardiovascular disease. WHO estimated that bacterial AMR was directly associated with 1.27 million deaths worldwide in 2019 [20]. According to the CDC, each year, more than 2.8 million antimicrobial-resistant infections occur in the USA, resulting in over 35,000 deaths. The economic burden of AMR in the USA is estimated to be $55 billion annually [21]. Additionally, it is estimated that AMR could result in an economic loss of 1–3.4 trillion USD due to medical expenses in the USA alone by 2030 [17,20]. The misuse and overuse of antibiotics in animals, including equines, have significantly contributed to the transmission of antibiotic-resistant bacteria [22]. Antibiotic-resistant pathogens such as *Escherichia coli* that produce extended-spectrum beta-lactamases (ESBL), methicillin-resistant *Staphylococcus aureus* (MRSA), antibiotic-resistant *Rhodococcus equi*, and multidrug-resistant *Salmonella* have all been repeatedly detected in horses [23,24,25].

AMR in equines has been garnering increasing attention in the last 20 years and has become a looming threat to the equine industry [18]. AMR results in a reduced efficacy of available antimicrobials, leading to increased treatment failures, severe infections, and reliance on more expensive alternatives [26]. The rising rate of AMR in the equine industry will create critical challenges in livestock and human health, as horses serve as reservoirs for zoonotic bacterial diseases [27]. Additionally, the emergence of multi-drug-resistant (MDR) bacteria exacerbates the situation and complicates treatment. MDR pathogens are those bacteria that are resistant to three or more classes of antibiotics. Several studies have reported the zoonotic transmission of MDR pathogens from equines [28,29,30]. For example, between 2012 and 2016, there was a reported increase in resistance to trimethoprim-sulfamethoxazole in *Streptococcus* spp. and *S. equi* in France [31]. Another report from France identified that the prevalence of MDR remained above 18% and 22.5% for *Staph. aureus* and *S. equi*, respectively [32]. Similarly, a retrospective epidemiological study on antimicrobial-resistant *Staphylococcus* spp. that were isolated from equine samples in Kentucky, USA, between 1993 and 2009 revealed that out of 1711 samples, 66.3% were resistant to at least one of the antibiotics tested, and 25% of the isolates were found to be MDR pathogens [33]. In another study conducted by Chung et al., out of 3078 samples collected from national racetracks in Korea, 4.6% were positive for *E. coli*, out of which 21% of the isolates were resistant to at least one antibiotic tested and 13.3% of the isolates possessed MDR [34].

Therefore, it is critical to assess the prevalence of AMR in equine populations and devise strategies to alleviate its impact. In this review, we discuss the history and types of antimicrobials used in horses, their effects on equine health, and the regulations and guidelines that are used to govern the use of antibiotics in veterinary practice. We also focus on the current status of AMR in horses, particularly highlighting its public health importance and the transmission dynamics between horses and other animals. This underscores the significance of adopting a One Health approach to combating AMR within the equine industry. Additionally, we provide insights into various bacterial infections in horses, their AMR profiles, epidemiological status, and zoonotic potential, along with possible recommendations for combating drug-resistant bacterial infections in humans and animals.

## 2. Equines Are a Crucial Component of One Health

According to the WHO, “One Health” is an integrated approach to balancing human health, animal health, and environmental interfaces [35]. Horses are vital to One Health as they influence zoonotic disease dynamics, contribute to therapeutic practices, impact environmental health, and support economic stability. Among the various animal-assisted therapeutic practices, equine-assisted therapy (EAT) has gained popularity for its effectiveness in rehabilitation, particularly in improving coordination, balance, and strength, including 26 different medical conditions reported previously [36]. Recognizing and optimizing these roles can enhance the health and well-being of humans, animals, and ecosystems [37]. Horses’ involvement with humans and the environment creates pathways for the transmission of various AMR pathogens, making it imperative to consider strategies aimed at combating AMR within the One Health approach [38]. Horses also provide crucial income globally through meat production, transportation, and significant roles in the breeding and racing industries [39,40]. The international travel of horses for racing or exhibition has become increasingly popular, and many individuals regard it as a form of recreation [41]. Horses’ roles as working animals, livestock, and pets make them one of the closest animals interacting with humans, which increases the risk of zoonosis. It was reported that 46.6% of the zoonotic diseases transmitted by horses are bacterial [29]. Furthermore, the continuous shedding of such bacteria leads to environmental contamination of the water, soil, and air [42]. The AMR bacteria can be transmitted to and from horses through different routes, including (1) contact with any infected animal species, including horses [30], (2) drinking and ingestion of contaminated food and water [43,44], (3) through vectors such as flies, mosquitoes, and rodents [44], (4) contact with infected humans [45], (5) inhalation of dust from contaminated environments [46,47], (6) poor hygiene and sanitation [48], and (7) wound contamination or during surgery [49].

Some of the horse pathogens, such as *Rhodococcus equi*, can infect immunocompromised humans and lead to the development of respiratory lesions [50]. Additionally, *Staph. aureus* from horses can infect various parts of the human or animal’s body as a result of cutaneous exposure and, if not treated in time, can lead to severe systemic infection and potentially life-threatening conditions like pneumonia [51]. The transmission of potentially drug-resistant bacteria from an infected horse to farm workers has also been reported [24,52]. For example, Weese et al. reported that three people working in a foal nursery contracted skin infections caused by Canadian MRSA-5 [52]. Similarly, in 2013, Pelkonen et al. reported three cases of *S. zooepidemicus* in men working with horses in eastern Finland [30]. Horses carrying infectious bacteria can also contaminate the environment through fecal shedding, respiratory secretions, or direct shedding from the skin. Bacterial shedding from horses can circulate in the environment and infect several hosts [43,44]. Contaminated manure and slurry, waste water, or other contaminated utensils, including bedding and feed, can transmit bacteria to fresh water and soil [48]. This leads to the contamination of fresh produce and results in potential human infections [53]. Another route of transmission for AMR pathogens is by insects or rodents as carriers or reservoirs [44]. Vectors can transmit such infections directly from infected horses or contaminated environments [54]. Houseflies can transmit *Salmonella*, thus causing foodborne diseases [55]. Several infectious bacteria, including *E. coli* O157, MRSA, *Clostridium*, *Listeria*, and *Streptococcus*, can be transmitted through vectors [56,57,58].

**Figure 1:**
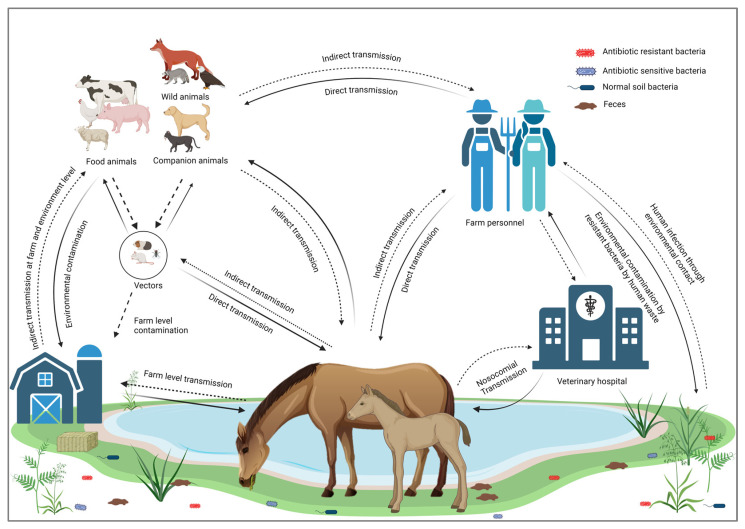
Transmission cycle of resistant bacterial infections to and from horses.

Horses are not only transmitting infectious bacterial pathogens but may also contribute to the transmission of their AMR profiles [59,60]. The excessive use of antimicrobial medications in horses induces a selective pressure that promotes the emergence of AMR bacteria [61]. Transmission of MDR bacteria poses a significant risk of spreading resistance genes to commensal bacteria [62]. Several studies have reported the transmission of AMR from horses to humans worldwide, highlighting this as a significant concern within the framework of One Health [63,64]. Transmission cycles of resistant bacterial infections from and to horses are illustrated in Figure 1.

## 3. Historical Usage of Antibiotics

The use of antibiotics in human and veterinary medicine has a long and intricate history. The era of the antibiotic revolution began with the discovery of penicillin by Sir Alexander Fleming in 1928 [65]. Tracing the development and widespread application of antibiotics, along with the simultaneous emergence of antibiotic resistance, offers valuable insights into the complex interplay between human innovation, animal applications, and microbial adaptations [66]. The use of antibiotics in animals’ dates back to the mid-20th century, following the purification of the first penicillin, called penicillin G, by Ernst Chain and Howard Florey in 1942 [67]. Similar to human medicine, the use of antibiotics revolutionized equine healthcare [11]. As antibiotic discoveries and usage spread worldwide, the use of antibiotics in the veterinary sector paralleled their use in human medicine. The early years (1900–1939) of antimicrobial drugs were referred to as the “Antiseptic Era” [68]. Antibiotics or antiseptic drugs could revolutionize the ability to treat and rid animals of infection by destroying targeted bacterial cells [16]. As decades of antibiotic usage continued, there were minimal restrictions regarding patent labeling or dosage [66]. People were able to obtain these drugs “Over the Counter” (OTC) for use in both humans and animals at any dosage or indication [69]. Generally, there were minimally controlled experimental studies to determine the appropriateness of these drugs for animal and human consumption. The discovery of antibiotics such as aminoglycosides and their widespread use during World War II for treating wounds, as well as later in animal production systems, marked the dawn of a new era of antibiotics. During the late 1940s and early 1950s, animal producers began implementing antibiotics such as tetracycline and chloramphenicol, neomycin, and aminoglycosides into their herd’s feed as growth promoters [70]. With the ability to implement antibiotics in a new way, the former “Antiseptic Era” soon turned into the “Wonder Era” [68]. For example, chlortetracycline was used to improve the growth rate of animals [71]. The “Wonder Era” came to an end in the late 1960s as new drugs started to emerge and become analogs [68,72]. The 1970s launched a “New Analog Era” regarding antimicrobial drugs [68] and the inclusion of antimicrobials into the feed of production animals and various livestock species. Applications of antimicrobials to animal feed saw an all-time high during the 1970s due to their ability to decrease the cost of production [70,73]. In 1980, the Federal Drug Administration (FDA) implemented a new regulation that restricted “medically important” antibiotics to be used in livestock and food-animal production, which had to be approved by a veterinary professional before use [74]. This regulation coincided with the emergence of MRSA [68], which became notable in the antimicrobial field not only for its AMR but also for its zoonotic significance. Consequently, this led to a movement to restrict antimicrobial use in animal production [75]. At the beginning of the 1990’s, the FDA revisited the concept of medical antibiotic usage [75], and in 1993, this agency released a statement requiring that all new applications for medically important antimicrobial products must be either “Veterinary Feed Directive” (VFD) or “prescription products” [74]. A Veterinary Feed Directive (VFD) is a written authorization from a licensed veterinarian for the use of an antimicrobial drug in animal feed, distinct from prescription drugs regulated by state pharmacies, which must not be mixed into feed or used off-label [76]. As antimicrobial usage had been widespread for decades at this point, concern for AMR began to rise. Governing bodies provided guidance for assessing potential AMR throughout the drug approval process [68,77]. Guidance for Industry #152, which was publicized in 2003, was the first presentation on guidelines for AMR within the drug approval process [74].

At the beginning of the 2010s, the FDA published its very first annual summary of all antimicrobial drugs that are sold or distributed with the intent of being used on food animals. For example, Guidance for Industry #213 provided antimicrobial and production sponsors with details on how to align their marketed products with the recommendations put forth by the FDA under Guidance for Industry #209’s protocols for a three-year treatment plan [78]. In 2012, the FDA published a draft regarding VFD’s proposed protocols; however, it was not until 2015 that the final rule regarding VFD’s was implemented. This ruling allowed veterinarians to be the final deciding factor on how VFD’s are able to be included in an animal’s feed, thus finalizing the move of OTC to VFD [74]. As AMR concern increased, new judicial constraints against antimicrobial drugs developed. Veterinarians support proper diagnosis to better create targeted treatments to ensure positive antimicrobial stewardship and preserve their efficacy [75]. Potential alternatives include phage therapy, probiotics, peptides, small molecules, vaccines, and immunomodulators to complement or replace antibiotics in human and veterinary medicines, which will help address the global health challenge associated with AMR and safeguard animal welfare [79,80,81]. In 2016, the first alternative antimicrobial drug (Imrestor) was approved in the USA and Canada [74]. In 2020, the FDA Center for Veterinary Medicine (CVM) released the National Antimicrobial Resistance Monitoring System (NARMS) Strategic Plan that runs from 2021–2025 [82]. This plan is centered around the concept of One Health, in which collaboration on a local, regional, national, and global level must occur to reach their outlined goal of “optimal health” for combating AMR [74]. Collaboration between veterinarians, researchers, regulatory agencies, and horse owners will be essential in implementing effective strategies to combat AMR in equine medicine. Opportunities for research, community education outreach, and policy enhancement will continue to arise in the future as AMR is at the forefront of animals and humans.

## 4. Regulations and Guidelines for Antibiotic Usage in Veterinary Practice

AMR poses a significant global threat that impacts both humans and animals. Within veterinary medicine, the use of antibiotics plays an important role in safeguarding animal health and welfare. However, the development of antibiotic resistance due to the misuse or overuse of the drugs has compromised treatment efficacy and poses current and potential concerns for biosecurity in the future. To address these issues, regulatory bodies and organizations have been established to implement stringent regulations and guidelines to govern the use of antimicrobial drugs in veterinary medicine. Implementing these guidelines and regulations aims to promote responsible antimicrobial stewardship, mitigate the development of AMR, and safeguard antimicrobial drug effectiveness for the future of both the animal industry and human populations. The USA “National Action Plan for Combating Antibiotic-Resistant Bacteria (CARB)” was established during 2020–2025 [83]. This strategy aims to: (1) reduce the growth of resistant bacteria and prevent the spread of resistant illnesses; (2) boost national One Health surveillance efforts to combat resistance; (3) advance the development and use of rapid and innovative diagnostic tests for the identification and characterization of resistant bacteria; (4) promote antibiotic stewardship and encourage responsible antibiotic use; (5) promote prevention, surveillance, control, and antibiotic research and development; and (6) foster national and international collaborations and partnerships among stakeholders [83]. 

**Figure 2:**
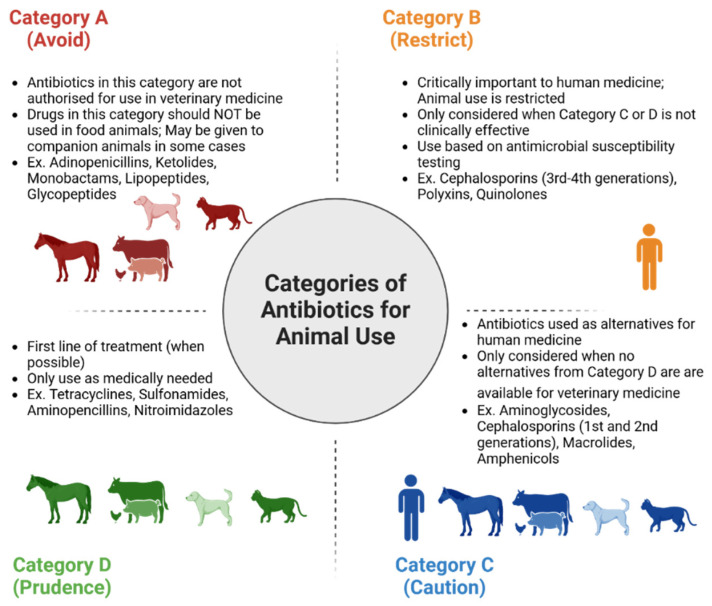
Categorization of antibiotics according to the European Medicines Agency’s Antimicrobial Advice Ad-Hoc Expert Group (AMEG) that are used in humans and animals. Antibiotics were divided into four categories: A, B, C, and D.

In 2022, the European Medicines Agency’s Antimicrobial Advice Ad-Hoc Expert Group (AMEG) designated antimicrobial drugs into four categories: A, B, C, and D. Category A refers to antimicrobials that should be “avoided”, as they are not authorized by the European Union (EU) for use in food or companion animals [79]. Category B refers to antimicrobials that are critically important in human medicine, such as quinolones, 3rd and 4th generation cephalosporins, and polymyxins, and have restrictions for use within veterinary medicine [79]. Category C drugs are to be used with “caution”, and it encompasses antimicrobials in which there are alternatives in human medicine but a limited number of alternatives for veterinary medicine. Category C drugs should only be pursued when Category D drugs are unattainable/resistance against them. Category D antimicrobials are known as “prudence” drugs and are the first line of antimicrobial treatments. Category D drugs are not intended to be used excessively or for long treatment periods [79]. Different categories of antimicrobials are shown in Figure 2.

There are a few principles to consider before using antimicrobials. These include: (1) Antimicrobial treatment should only be started if a bacterial infection is proven or strongly suspected, after a thorough clinical examination and assessment, including supportive tests. The veterinarians should consider that the animal’s immune system might be able to fight the infections without antibiotic treatment. (2) Determine whether a topical antiseptic or systemic antimicrobial treatment is better suited to treat the infections. (3) Ensure that any underlying conditions are treated to minimize the risk of repeated infections. (4) Choose the appropriate antimicrobial agent and mode of administration based on absorption, tissue penetration, activation potential, and toxicity in the patient. (5) Category A antimicrobial drugs should never be used in food-producing animals. (6) Category D drugs are the first line of antimicrobial defense, followed by Category C drugs if there are no alternatives, and use narrow-spectrum treatment wherever possible. (7) Only use Category B agents if no other suitable agent is available based on culture and susceptibility test results, or in severe disease cases. (8) Consider exposing the animal patient to the least amount of antimicrobials when selecting treatment for polymicrobial infections. (9) Consider using cytology and other diagnostic tests to determine infection remission and treatment duration. (10) When possible, reduce therapy to a lower-category agent based on culture and susceptibility data [79].

## 5. Practical Applications of Antimicrobials in Equines

In adult horses, veterinarians follow specific guidelines for the use of antimicrobials based on the condition being treated, the geographical location, and the sensitivity patterns of the involved bacteria in that region. Antibiotics are not routinely used for conditions such as hoof abscesses, non-serious wounds, aseptic joint injections, routine castrations, and many surgeries. However, perioperative treatment with penicillin is often administered for 24 h for minor surgeries. For mild to moderate dermatitis or folliculitis, systemic antibiotics are not needed, and topical treatments are often effective. In cases of lymphangitis, antimicrobials are required if their sepsis supervenes. A horse exhibiting symptoms of cellulitis requires antimicrobial and anti-inflammatory drugs, with doxycycline, which is recommended as the first line of treatment, and gentamicin/penicillin as alternative options, along with the TAT (Tetnus-Antitoxin). Severe dermatitis or folliculitis necessitates the use of trimethoprim, sulphadiazine, procaine penicillin, or doxycycline to manage subcutaneous bacterial infections [79].

For respiratory diseases like cough, tracheal aspirate should be analyzed to detect potential bacteria, with polymerase chain reaction (PCR) serving as the gold standard for microbe detection. The first line of treatment includes trimethoprim sulphadiazine (TMS), procaine penicillin, or doxycycline for common bacterial infections. In cases of bacterial pleuropneumonia, a broad-spectrum treatment such as penicillin, gentamicin, and metronidazole is recommended. Broad-spectrum antibiotics are also advised for severe cases of toxic diarrhea in horses, coupled with intensive supportive care [79]. Again, bacterial sensitivity should always guide the treatment of individual animals and conditions, when available.

In mares, no antimicrobial treatment is required for normal, routine breeding. A physiological breeding-induced endometrial inflammation is triggered by semen as well as contaminating bacteria, but close to 90% of all mares are fully capable of eliminating all bacteria and inflammatory products within 24–36 h after breeding through the local innate immune system and uterine contraction [84,85]. The small portion of brood mares that fail to clear bacteria after breeding are best treated with uterine lavage and/or ecbolic drugs before the bacteria establish an infection [86]. In addition, good hygiene practices, like perianal cleaning, will reduce bacterial contamination [79]. The most common bacteria associated with chronic bacterial endometritis in cycling mares are *S. equi* spp. *zooepidemicus*, *E. coli*, *Staphylococcus* spp., and *P. aeruginosa*, with *Klebsiella pneumonia* less commonly cultured from mares [87]. These bacteria enter the uterus in a free-floating planktonic state, which makes them vulnerable to the host immune system as well as exposure to local or systemic administration of an appropriate antimicrobial. While the bacteria are generally sensitive to a variety of antibiotics in their planktonic state, their failure to respond to treatment may be due to the presence of dormant bacteria or the development of a bacterial biofilm community in the endometrium. Dormancy allows bacteria to escape antimicrobial treatment, and the microorganisms do not generate an inflammatory reaction, making them difficult to detect with standard diagnostics. Diagnostic strategies and treatment protocols have been described for endometritis caused by dormant bacteria, and these should be followed when the condition is suspected [88,89]. Bacteria that are allowed to adhere to the endometrial surface may form micro-colonies, develop a complex structure, and switch into a biofilm community [90]. While bacteria on the surface of a biofilm are exposed to sufficient concentrations of antibiotics, there is a gradually reduced diffusion through the biofilm matrix that leads to a decreased concentration of the antibiotics within the biofilm community. This provides an excellent opportunity for bacteria to develop resistance to antimicrobial drugs. In addition, genetic alterations associated with antibiotic resistance are easily transmitted among bacteria within the bacterial biofilm community [91].

Placentitis is a leading cause of late-term pregnancy wastage in mares [92,93]. Placentitis could be classified based on morphological lesions (site and distribution) and suggested pathogenesis into four forms: ascending, diffuse (hematogenous), multifocal, and focal mucoid (nocardioform) [93]. Ascending placentitis, the most frequent form, arises from an ascending infection (mainly *S. zooepidemicus*), which gains access to the cervical star region (caudal pole of the chorioallntois) [92,94,95]. Other bacterial agents have been associated with this form of placentitis, including *E. coli*, *S. equisimilis*, *K. pneumoniae*, and *P. aeruginosa* [92,93]. Focal mucoid placentitis is associated with gram-positive branching bacilli (mainly *Amycolatopsis* species and *Crossiella equi*). Diffuse and multifocal placentitis are the less common forms and involve the hematogenous spread of microorganisms to the placenta, often due to *Leptospira* infection [92,93]. Early diagnosis and treatment of equine placentitis could improve pregnancy outcomes. In ascending placentitis, transrectal ultrasonographic detection of placental abnormalities such as an increase in the combined thickness of the uterus and placenta (CTUP), along with separation between the chorion and endometrium, is considered diagnostic for this form [92]. The standard treatment includes long-term antimicrobials, progestins, and anti-inflammatory drugs [96]. However, reports on pharmacokinetics in mares with ascending placentitis show some antimicrobial drug concentrations at lower concentrations in the fetal fluids than in circulation, and over 60% of mares receiving treatment for ascending placentitis still harbored the causative bacteria in the uterus after delivery of a foal [97,98]. This suggests that current treatment practices suppress, rather than eliminate bacterial growth from affected placentae, potentially increasing the risk of developing antimicrobial resistance [99].

In foals, several health issues may occur early in their lives, including umbilical infections, patent urachus, septic arthritis/physitis, respiratory disease, and diarrhea [100]. Broad-spectrum antimicrobial drugs, like trimethoprim and sulphadiazine, are often prescribed to treat patients with clinical signs of umbilical infection, and chlorhexidine (0.5%) for external treatment. Before a broad-spectrum antimicrobial is prescribed for septic arthritis/physitis, an arthrocentesis, blood sample for culture, and sensitivity (C and S) should be completed [101]. In the case of respiratory diseases, for 1–5-month-old foals, common causes of bacterial pneumonia include *R. equi* and *S. zooepidemicus* [102]. Antibiotics are typically not used for sub-clinical *Rhodococcus* infections, as these often resolve spontaneously without antimicrobial therapy. However, for clinical manifestations of rhodococcal pneumonia accompanied by thoracic abscesses, macrolides combined with rifampicin are the recommended treatment [103]. As for young stock horses, who are 1–3 years old, treatment for respiratory conditions will often require penicillin, trimethoprim, or sulfadiazine to be treated. Young foals who experience diarrhea are often treated with broad spectrum antimicrobial drugs. As for weanlings experiencing diarrhea, after diagnostic testing, the implementation of doxycycline has been recommended for treatment, but as always, individual treatments for specific conditions should be guided by molecular diagnostics and antimicrobial sensitivity to antibiotics.

## 6. Drawbacks of Antibiotic Usage in Equine Production

Antibiotics have become essential in veterinary medicine for their ability to treat infectious bacteria [104]. Although these drugs have shown undeniable benefits throughout history to producers and stakeholders, antibiotics demonstrate adverse effects on equine health [11]. In addition to the emergence of AMR, some adverse effects caused by the use of antimicrobial drugs in horses include loss of appetite, allergic reactions, and antibiotic-associated diarrhea (AAD) (Table 1) [105]. These drawbacks have negative consequences that reach not only the sector of animal production but also global public health [106].

### 6.1. Disruption of Normal Microbiota

The administration of antimicrobial drugs in horses can cause an imbalance of the beneficial gut microbiota of the host and interfere with their composition and function [124]. These gut microbiota play a crucial role in preventing pathogen colonization, regulating gut immunity, the digestion of essential nutrients and bioactive metabolites, and the generation of energy homeostasis within the environment [104]. The gut microbiota also plays an important role in the gut-brain axis, as it influences the brain’s behavior and functional efficiency [124,125]. Similarly, in equines, the intestinal microbiota plays a significant role in immune function, gut-brain axis and behavior, conditions like diabetes and obesity, nutrient digestion, production of short-chain fatty acids, and providing barrier functions [126]. Thus, disruption of normal gut microbiota can lead to an inability of efficient digestion of nutrients, AAD that is associated with severe diarrhea, rapid dehydration, electrolyte deficiency, competition with pathogens for substrate intake, reduced colonization resistance, endotoxemia, and, in severe cases, death [127,128]. A recent study demonstrated a dramatic decrease in the abundance of *Lactobacillus* spp. in horses administered with potassium penicillin/gentamicin sulfate, ceftiofur crystalline free acid, and trimethoprim/sulfamethoxazole (SMZ). The authors also demonstrated an increased abundance of *C. difficle* or *C. perfringens* 28 days post-administration of antibiotics [104]. Similarly, intravenous administration of four treatment groups, including enrofloxacin, ceftiofur sodium, and oxytetracycline, demonstrated an alteration of microbial diversity and quantity in all the treatment groups as compared with the 0.9% saline control group [127]. Additionally, Collinet et al. observed diarrhea in horses the second day of antibiotic administration with a decrease in relative abundance of *Eubacterium*, *Lachnospiraceae* AC2044, *Ruminococcus*, and *Saccharofermentans* groups of intestinal bacteria, which play an important role in plant cell wall degradation and fermentation in the gut [129]. In another study, Costa et al. reported that the administration of trimethoprim TMS caused marked changes in the fecal microbiota at a higher taxonomic level with decreased richness and diversity, with a significant decrease in the Verrucomicrobia phylum in the microbiota population [126]. Therefore, the disruption of the microbiota caused by an antibiotic treatment can severely impact the overall health of the affected horse. Major alterations in the microbiota composition can lead to the colonization of multi-drug-resistant pathogens in the equine gut [130].

### 6.2. Selection Pressure for the Suitable Treatment of Certain Illnesses

As bacteria develop resistance to antibiotics, the resulting negative impact on clinical and therapeutic outcomes has become increasingly evident [131,132]. As resistance increases, so does the rate of treatment failures, morbidity and mortality rates, lengths of hospitalizations, and treatment costs [133]. This necessitates the development of new alternative antimicrobial drugs, the cost of which is significantly high [132]. One of the critical contributing factors to the rise and proliferation of AMR bacteria is selection pressure [134]. Selection pressure is driven by the inappropriate use or overuse of antibiotics, causing bacteria exposed to antimicrobial drugs to undergo rapid genetic changes [135]. These genetic adaptations enable the resistant bacteria to proliferate and outgrow their susceptible counterparts, which are eliminated. As a result, resistant strains become predominant [136]. In a cluster-randomized experiment in Ethiopia, 12 groups received mass azithromycin therapy for children aged 1–10 at 0, 3, 6, and 9 months, while 12 control groups received the antibiotic only after 12 months. This mass administration led to a dramatic increase in azithromycin-resistant *S. pneumoniae* among treated children, from 3.6% at 0 months to 46.9% at 12 months, compared with only 9.2% in the control group [136]. This suggests that the overuse or improper use of antibiotics generates strong selection pressure, which favors the survival and proliferation of resistant bacteria [137]. Similarly, in another randomized trial conducted by Keenan et al. involving 120 children in the Matameye district of the Zinder region in Niger, it was demonstrated that the annual or biannual mass administration of azithromycin led to an increased prevalence of macrolide resistance determinants, *ermB* and *mefA/E,* in nasopharyngeal swabs containing *S. pneumoniae*. Specifically, the prevalence was 29.4% higher in the biannual group compared with the annual group after a period of 24 months, highlighting the role of selection pressure on the development of antibiotic resistance [138]. A prime example of selection pressure for certain kinds of illnesses is determining what antimicrobial drug to use for respiratory illnesses in the face of bacterial resistance [18]. For instance, in foals, *R. equi* pneumonia is a prevalent condition typically addressed with macrolide combinations like erythromycin, azithromycin, or clarithromycin in conjunction with rifampicin [121]. However, prolonged and widespread use of these drugs has significantly heightened the selective pressure leading to antibiotic resistance [135]. *R. equi* isolates collected from environmental samples and up to 40% of pneumonic foals have exhibited acquired resistance against macrolides and rifampicin [139]. Hence, selection pressure presents a severe disadvantage to antibiotic usage in both humans and animals. Resistance caused by antibiotic overuse or misuse accelerates the growth and spread of antibiotic-resistant bacteria, posing a substantial threat to effective infection control and public health worldwide.

## 7. Epidemiology of AMR Infections in Equines

Bacterial infections are one of the major causes of production and economic losses within the equine industry [140,141]. However, the growing threat of AMR complicates treatments, and antibiotics may no longer be effective to safeguard equine health [142]. Antimicrobials being used to treat equine infections/diseases and their reports of AMR worldwide are listed in Table 1. To help understand the challenges and develop effective strategies for treatment and prevention, we need to understand the epidemiology of AMR bacteria in equines.

### 7.1. Bacterial Pathogens Affecting Gastrointestinal Tract of Horses

#### 7.1.1. *Salmonella* Infections

Salmonellosis poses a significant health concern in equine and is frequently observed in veterinary hospitals and on breeding facilities [143,144,145,146,147,148,149,150,151,152,153,154,155]. It has been reported that up to 70% of normal, healthy horses are infected with *Salmonella* worldwide [146,151,156,157]. However, the reported fecal shedding of *Salmonella* by healthy horses in the USA was only 0.8% in one study from 2000 [158]. *Salmonella* in adult horses is usually characterized by fever and diarrhea, while in foals, the infections often result in septicemia [159]. The co-infection of the equine digestive system caused by *Salmonella,* together with rotavirus and enterotoxigenic *Bacteroides fragilis* infection, can worsen the horse’s condition [160,161]. Moreover, subclinical infections can become clinical due to parasitic infections, hot weather, and excessive training [162]. Epidemiological reports indicate that the development of disease in equine populations significantly depends on several factors, including bacterial characteristics, host immunity, and the environment [163]. Additionally, stress conditions such as transportation can affect *Salmonella* infections [164]. The transmission of bacteria to healthy horses mainly occurs through the consumption of contaminated water and pasture [165]. Additionally, transmission can occur through direct or indirect contact with contaminated and damaged flooring, non-cleanable surfaces, and unsealed concrete and wood [166]. Furthermore, farm workers, common equipment, including rectal thermometers and water or feed buckets, can act as sources of infection [122]. Farms housing multiple animal species with horses, including cattle, dogs, cats, and poultry, have demonstrated a high risk of cross-species transmission [167,168,169,170]. Horses housed in their natural environment are reported to exhibit a significantly reduced prevalence of *Salmonella* shedding, as shown by comparative surveys of hospitalized and naturally housed horses [146,151]. *Salmonella* serotypes such as *Salmonella* Typhimurium, *Salmonella* Newport, *Salmonella* Javiana, and *Salmonella* Anatum are the most common serotypes reported in the USA [171]. Leon et al. [172] reported *Salmonella* Newport as the most frequent serotype in Texas, which agreed with some other studies [49,173,174]. However, several studies have reported that *Salmonella* Typhimurium is the most dominant cause of salmonellosis in horses [175,176,177].

*Salmonella* infections are generally self-limiting [178]. However, in severe cases, the treatment typically relies on antibiotics [179]. *Salmonella* infections in horses are normally treated with chloramphenicol and trimethoprim-sulfonamides [180]. However, there have been several reports of the development of AMR in these antibiotics, which has impacted their effectiveness in controlling salmonellosis in equines in the USA [181]. Between 2001 and 2013, multidrug-resistant (MDR) *Salmonella* was detected in 57% of the isolates. Specifically, 21.6% of the isolates were resistant to gentamicin, 41.2% were resistant to ceftiofur, 59.3% were resistant to chloramphenicol, and 26.7% were resistant to trimethoprim-sulfonamides. However, only 1.0% of the isolates were resistant to enrofloxacin [49]. Additionally, from 2001 to 2013, *Salmonella* isolates from hospitalized horses at Cornell University exhibited AMR to several antibiotics: amoxicillin-clavulanic acid (29%), ampicillin (45.5%), cefazolin (42.2%), cefoxitin (27.5%), ceftiofur (37.3%), chloramphenicol (45.2%), and tetracycline (46.1%) [49]. *Salmonella* isolated from diagnostic samples of horses and submitted to veterinary diagnostic laboratories in four states (Arizona, Missouri, North Carolina, and Tennessee) demonstrated resistance to ampicillin (20%), chloramphenicol (20%), and sulfamethoxazole (20%) [182]. Furthermore, in Europe between 1993 and 2000, similar trends in AMR for the seven antimicrobials were detected. The highest resistance was observed against tetracycline (53%), followed by ampicillin (34%), SMZ (21%), kanamycin (12%), gentamicin (6%), and enrofloxacin (5%), and none of the isolates were resistant to ceftiofur [183]. In the United Arab Emirates, *Salmonella* isolates detected from healthy and diseased animals between 1996 and 2009 possessed resistance to ampicillin (25%), cefoxitin (7.9%), chloramphenicol (9%), ciprofloxacin (17%), gentamicin (20%), kanamycin (12%), nalidixic acid (28%), streptomycin (30%), and trimethoprim-sulfamethoxazole (15%) [170]. Almost similar reports were observed in equine isolates from India between 1982 and 1996 [184]. Serotype dominance was also noted in AMR reports, where one study demonstrated that isolates of the serotype *Salmonella* Anatum showed the greatest resistance, followed by those of *S.* Rubislaw and *S.* Braenderup [172]. In contrast, Cummings et al. (2016) identified *S.* Newport, *S.* Oranienburg, and *S.* Typhimurium as the most resistant serotypes [49]. Another study conducted at a veterinary hospital in Florida (USA) reported that *S*. Java, *S*. Typhimurium var. Copenhagen, *S*. Javiana, and *S*. Newport were the predominant serotypes in terms of resistance [174]. A similar study by Van Duijkeren et al. in the Netherlands found that *S.* Typhimurium was the most resistant serotype [183].

Taken together, the growing problem of AMR in *Salmonella* underscores the urgent need for new approaches to treatment and prevention. Control of *Salmonella* in an equine setting requires stringent biosafety and biosecurity measures. Implementing comprehensive hygiene protocols, including regular disinfection of facilities, proper management of animal waste, and isolation of infected animals, can significantly reduce the risk of infection transmission. Additionally, staff should be trained in proper handling and sanitation practices to minimize any potential cross-contamination. Continuing surveillance efforts are crucial to monitoring resistance patterns and adapting control strategies accordingly.

#### 7.1.2. *E. coli* Infections

*E. coli* are ubiquitous organisms, with most strains being commensal microorganisms in mammals [185,186]. However, some strains can cause diseases in both humans and animals [185,187]. Horses are exposed to *E. coli* shortly after birth, often through suckling or exposure to environmental objects [188]. The gastrointestinal tract of horses, specifically the intestines, are the most common reservoirs for *E. coli.* These bacteria can be extraintestinal [189]. In foals, *E. coli* can invade the intestinal mucosa, resulting in enteritis, colitis, or both. This is known as colibacillosis. Colibacillosis is typically caused by antibiotic-associated dysbiosis [189]. The transmission of *E. coli* primarily occurs when a healthy horse ingests contaminated water, pasture, or encounters feces or farm instruments that have been contaminated with fecal matter [190]. Horses can serve as reservoirs for diarrheagenic *E. coli* and can potentially contribute to human infections [191]. Enteropathogenic *E. coli* (EPEC) and Shiga toxin-producing *E. coli* (STEC) have frequently been reported in horses worldwide, despite their prevalence being less than 1% among other *E. coli* serotypes [192,193,194,195]. Infections caused by *E. coli* in horses are typically transient and infrequent [193]. Despite the rarity of *E. coli* in equine manure, implementing good agricultural practices, such as regularly removing feces from sheds or fields, can significantly reduce contamination and transmission of the bacteria to healthy horses [193].

Infections with *E. coli* are typically treated with antibiotics such as tetracyclines, sulfonamides, cephalosporins, macrolides, and aminoglycosides. However, the bacteria have been reported to have obtained resistance against these antibiotics [196,197]. Moreover, the ubiquitous presence of bacteria in the gastrointestinal tract along with recurrent exposure to antibiotics during treatment are some of the contributing factors for the increased selection pressure leading to the genomic evolution of the bacteria and the development of AMR [198]. Horses have been reported as asymptomatic carriers of highly pathogenic *E. coli O157* strains and have been documented as major transmitters of the infection to both livestock and humans. [199,200]. This species often encounters AMR, particularly β-lactam antibiotics, which is a significant concern. *E. coli* exhibits intrinsic resistance to penicillin due to its inability to effectively traverse its outer membrane [201]. Additionally, substantial acquired resistance to other β-lactams exists, primarily due to the production of inactivating enzymes such as TEM-1, TEM-2, and SHV-1, as well as *AmpC* β-lactamases, which are encoded by various *bla* resistance genes [202,203]. These genes are primarily encoded in plasmids or mobile genetic elements that are responsible for dissemination to other isolates [204].

AMR in *E. coli* poses a significant threat to horse welfare. In the USA, the prevalence of AMR *E. coli* in horses was reported at 11.9% in 2023 [205]. The most prevalent resistance was observed against sulfonamide drugs, with 10.3% resistance to sulfisoxazole and 9.3% resistance to SMZ [205]. This finding aligns with previous estimates for horses in community settings. The studies on AMR in *E. coli* from non-hospitalized horses have often focused on small, regional samples or were conducted in countries outside the USA [118,206]. Resistance to sulfonamides, including SMZ, is commonly observed in *E. coli* isolated from horses, likely due to the frequent and overuse of these antimicrobials in both hospitalized and non-hospitalized settings [207]. In a study conducted in France, 11% of *E. coli* isolates from equines were identified as MDR, with reported resistance rates of 18% to ceftiofur, 25% to gentamicin, and 26.2% to SMZ [31]. Similarly, another study from Thailand reported resistance rates of 15.2% to ampicillin, 12.1% to cephalothin, 12.1% to tetracycline, and 9.1% to SMZ in 2022 [208]. In conclusion, AMR complicates treatment regimens for *E. coli* infections in equines, particularly foals, and these findings highlight the necessity for vigilant surveillance, judicious antimicrobial use, and the development of effective treatment strategies to safeguard equine welfare and mitigate the spread of resistant strains.

#### 7.1.3. *Clostridium* Infections

*C. difficile* and *C. perfringens* are identified as major contributors causing acute enterocolitis in horses [209]. The first report of *C. difficile* infections in equines dates back to 1984, in the Potomac River area [210]. The infection of *C. difficile* and *C. perfringens* has been associated with diarrhea or acute colitis in horses, where the prevalence ranging from 5% to 90% has been documented [211,212,213,214,215]. The transmission of *Clostridium* occurs through the ingestion of vegetative cells or *Clostridium* spores from infected horses, contaminated environments, or potentially other animal species, as well as humans [216]. These bacteria will initially colonize the gastrointestinal tract of the host at low levels, as the host remains an asymptomatic carrier [217,218]. Subsequently, the bacteria proliferate, especially in the presence of predisposing factors like gut dysbiosis [219]. Major factors contributing to *Clostridium* infections in horses include hospitalization and antibiotic treatments [219,220]. Most of the antibiotics can lead to diarrhea and enterocolitis in horses, particularly when opportunistic enteropathogenesis occurs or other risk factors are present [221,222]. Antibiotics can disrupt the distribution and abundance of the gut microbiota, leading to AAD [223]. This disruption can lead to the proliferation of pathogenic bacteria, including *C. perfringens* and *C. difficile* [224]. β-lactam antibiotics, erythromycin, SMZ, rifampicin, clindamycin, and gentamicin are commonly implicated in causing AAD in horses [211,225]. In addition, hospital environments may heighten the potential exposure of the host to *Clostridium* due to contaminated instruments. Meanwhile, factors such as dietary changes, pre- and post-operative fasting, and antibiotic use during hospitalization could also contribute to the development of *Clostridium*-associated infections in horses [217,218].

*C. difficile* is one of the most important causes of enterocolitis in horses [221]. Epidemiological reports have revealed that healthy adult horses carry *C. difficile* at significantly reduced rates, whereas foals typically harbor the bacterium more frequently [211]. Horses, both as adults and foals, are susceptible to *C. difficile* enteric disease, which can develop as early as the first few days of life [226]. In Sweden, a study conducted in 2003 revealed that *C. difficile* was isolated from 29% of healthy foals under the age of 14 days old and only 0.6% of foals aged more than 14 days old [211]. In hospital settings, the prevalence was observed at about 31% in foals and 19% in mares [227]. The same trend was observed in a 2019 study conducted in Saudi Arabia, where *C. difficile* was found in 7.1% of healthy foals and 22.5% of diarrheic foals, all of whom were under 2 months old. Notably, *C. difficile* was not isolated from any older foals [228]. *C. difficile* produces toxins A (TcdA) and B (TcdB), which are well-established virulence factors of the bacteria [229]. In horses, metronidazole is commonly used in combination with zinc bacitracin for the treatment of colitis; however, nearly all *C. difficile* isolates are resistant to these treatments [230,231,232]. The strains of *C. difficile* resistant to metronidazole are regarded as more aggressive in terms of the severity of the disease than those sensitive to the antibiotic [233]. Similarly, a study in Sweden reported 100% resistance of *C. difficile* to trimethoprim/sulfamethoxazole and bacitracin. The authors also found resistance to erythromycin (26.9%) and rifampicin (26.9%) in some isolates [211]. Another study in Belgium reported 100% resistance to clindamycin, gentamicin, and ceftiofur and intermediate resistance to penicillin [233]. Furthermore, a study conducted between 1998 and 1999 in Ontario, Canada, demonstrated that all isolates from foals were susceptible to metronidazole. However, 95% showed resistance to cefotaxime and bacitracin, and 75% were resistant to SMZ [232].

*C. perfringens* is known to cause enterotoxemic diarrhea in foals, which can cause necrotic lesions in the gut mucosa [206,234,235]. *C. perfringens* was thought to be one of the commensal gut organisms; however, low prevalence was observed in adult horse feces, ranging from 0 to 8% [206]. A study conducted in Florida between 2003 and 2008 revealed that C. *perfringens* is more frequently found in foals under one month old experiencing diarrhea, with a prevalence of 18% [212]. Another study, conducted between 2017 and 2020 at UC Davis and in Canada, found a 25% prevalence in diarrheic foals less than 30 days old [236]. The pathogenesis of enterotoxaemia caused by *C. perfringens* involves the production of various toxins, such as alpha (CPA), beta (CPB), epsilon (ETX), iota (ITX) toxins, enterotoxin (CPE), necrotic enteritis-like B toxin (NetB), and pore-forming toxin, NetF [237,238,239]. Seven genotypes have been observed in *C. perfringens*, where Genotype A, which makes up 85%, and Genotype C, ranging from 1 to 3%, commonly occur in *C. perfringens*-associated diarrhea in foals [235]. The clinical signs involve colic, lethargy, pyrexia, depression, and often bloody diarrhea. However, in some cases, neurological signs and sudden death may occur [237]. Like for *C. difficile*, metronidazole is commonly used to treat *C. perfringens* infections in horses [206,232]. However, there are limited reports of AMR in *C. perfringens* isolated from horses. A study conducted in Egypt between 2017 and 2018 reported 100% oxytetracycline-resistant isolates from foals [240]. Similarly, a study conducted in South Korea between 2009 and 2017 reported that 12% of amoxicillin/clavulanic acid-resistant isolates were detected, with 4% showing resistance to vancomycin and meropenem. These isolates were obtained from deceased horses and the soil surrounding their bodies [241]. The reports of AMR in *Clostridium* isolates from equines are relatively infrequent worldwide. However, the use of antibiotics contributes to the emergence of *Clostridium*-associated diseases, and the antibiotics commonly used for treatment are facing increasing resistance. Consequently, it is crucial to use antibiotics judiciously in foals to prevent AAD caused by clostridial pathogens and to mitigate the development of AMR.

#### 7.1.4. *Campylobacter* Infections

*Campylobacter* infections in horses, though less frequently discussed compared with other gastrointestinal pathogens, pose a significant threat to equine health [242]. *Campylobacter* species, particularly *Campylobacter jejuni* and *Campylobacter coli*, are well-known for causing enteric diseases in various animal species and humans [243,244,245]. The zoonotic potential of *Campylobacter* adds another layer of importance to understanding and managing these infections in equine populations [29]. *Campylobacter* is not generally considered a commensal bacterium in horses [246]. Horses can become infected with *Campylobacter* through various routes, including contaminated feed and water, direct contact with infected animals, or environmental exposure [247]. In foals, the infection can be particularly severe due to their undeveloped immune systems and greater susceptibility to dehydration and systemic complications [248,249]. Although *Campylobacter* spp. are infrequently found in horses, there is a growing consensus that routine monitoring for *C. jejuni* and *C. coli* is important, as these bacteria have the potential to cause chronic diarrhea in horses [250]. The occurrence of *Campylobacter* infections in horses ranges from 3% to 60% worldwide [247,251,252,253]. In 2010, a study conducted in New Zealand with healthy horses identified all *Campylobacter* isolates as *C. jejuni*, marking the first report of its kind in the country [247]. Similarly, another study conducted in the USA reported the isolation of *Campylobacter* spp. from a fecal sample of a sick 2-year-old horse with chronic diarrhea [250]. Furthermore, a study conducted by Atherton et al. identified *C. jejuni* as the causative agent of hemorrhagic enteritis in young foals under 6 months of age [249]. However, a study conducted in Britain and Ireland revealed a very low prevalence (<1%) of *Campylobacter* spp. in the feces of foals [254]. Additionally, several studies were unable to detect *Campylobacter* spp. in fecal samples from both healthy and diarrheic adult horses [255,256]. The complex isolation and detection techniques required for this organism make it challenging to report this pathogen.

Antibiotics such as erythromycin, tetracycline, gentamicin, and fluoroquinolones have been reported as effective treatments for *Campylobacter* infections [257,258,259,260]. The selection of the appropriate antibiotic and the duration of treatment can vary depending on factors such as the severity of the infection, the overall health condition, and the presence of any AMR. A study conducted by Selwet et al. demonstrated that tetracycline possessed a high susceptibility to *Campylobacter*; however, another study demonstrated resistance to tetracycline (6.25%). The same study also found that 78% of the isolates were resistant to ampicillin, and 44% exhibited resistance to erythromycin [261,262]. Similarly, a 2020 study conducted on 100 horses from southeastern Poland demonstrated that resistance to fluoroquinolones was 2.6% in *C. jejuni* and 40% in *C. coli* [253]. The challenge of AMR *Campylobacter* underscores the need for judicial antibiotic strategies and ongoing surveillance. Despite the infrequent reporting of *Campylobacter* spp. in horses, routine monitoring and an appropriate treatment strategy remain essential. Effective management depends on a thorough understanding of AMR patterns and strict antimicrobial stewardship to protect horse health and prevent the spread of resistant *Campylobacter* strains.

#### 7.1.5. *Staphylococcus aureus* Infections

*Staph. aureus* is a gram-positive coccus frequently present on the skin and mucous membranes of humans and animals, recognized for its ability to cause various infections [263]. *Staph. aureus* is typically a component of the normal equine microflora. However, it can cause infections when the skin or mucosal barriers are breached, allowing the bacteria to invade underlying epithelial tissues [114]. *Staph. aureus* infections frequently affect the skin, soft tissues, bones, and joints [264], causing skin abscesses, cellulitis, pneumonia, and occasionally systemic infections [264]. Transmission of the bacteria to healthy horses occurs by direct contact with the infected horses, in a contaminated environment, and through farm personnel [265]. These bacteria are categorized as coagulase-positive or coagulase-negative [266]. While coagulase-negative staphylococci have typically been considered non-pathogenic, some are recognized for causing opportunistic infections [267]. In equines, coagulase-negative species are the most common commensal staphylococci, with various species colonizing mucous membranes [268]. Although the carriage of coagulase-positive staphylococci is less common, these bacteria are known to cause multiple infections in horses [269,270].

The emergence of AMR in *Staph. aureus* is a growing concern, particularly with strains such as MRSA. This genus commonly exhibits resistance to various antimicrobial agents [271]. Nevertheless, resistance to the narrow-spectrum β-lactam methicillin is particularly significant, as it normally indicates resistance to all β-lactam medications [272]. MRSA remains the most extensively studied AMR bacterium in horses, likely due to its pathogenic potential and the risks associated with zoonotic transmission [273,274]. Initially, MRSA infections in horses were mostly seen in isolated incidents associated with veterinary hospitals. However, it is now more widely acknowledged as an emerging threat within the equine community [275,276]. MRSA infections have been recorded in North America [276,277], Ireland [278], Japan [279], Austria [280], and the United Kingdom [277]. In Europe, Canada, and North America, the prevalence of MRSA colonization in horses varies from 0% to 4.7% throughout equine farms [24,281,282,283], and between 2.9% and 10.9% in horses admitted to equine hospitals in Canada [284,285]. In an equine hospital setting in San Antonio, TX, USA, the prevalence was found to be as high as 16% [286].

Several reports indicate that MRSA infections in horses often originate in humans [287]. The human epidemic clone Multi-locus Sequence Type (MLST) ST8, also known as Canadian MRSA-5 or USA500, is responsible for the majority of MRSA infections in horses in Canada and North America [288]. This particular MRSA strain is believed to be adapted to horses due to its prevalence among other horses and farm personnel [288]. Recently, there have been reports of horses being colonized and infected with MRSA ST398, a strain associated with livestock [265,289]. The primary mode of transmission of infection within veterinary hospitals is through contamination on the hands of veterinary personnel [277]. In clinical cases, the treatment of MRSA in horses is predominantly based on antimicrobial susceptibility results, when available. To ensure that the selected treatment is efficacious and that no further resistance is developed, repeated testing should be considered during therapy [263,290]. MRSA isolates are considered resistant to all β-lactams, cephalosporins, and related compounds, regardless of the results of in vitro testing [291,292]. MRSA isolates occasionally exhibit apparent sensitivity to amoxicillin/clavulanate; however, clavulanate, which functions as a β-lactamase inhibitor, does not influence the β-lactam-resistant strains [293]. In addition, equine MRSA isolates exhibit widespread gentamicin resistance as well as variable resistance to tetracyclines, fluoroquinolones, and potentiated sulfonamides [278,280,282]. Most MRSA isolates, including those from horses, are susceptible to other antimicrobials, including vancomycin, linezolid, and teicoplanin. These antibiotics are frequently employed to effectively treat infections in humans [294]. However, these drugs are restricted to veterinary use [287]. In Canada, the reported MDR staphylococci were 83%, with resistance against enrofloxacin (75%), erythromycin (75%), tetracycline (10%), SMZ (95%), and gentamycin (95%) [295]. On the other hand, in the USA, 92.5% of oxacillin-resistant staphylococci (ORSA) were MDR, and the highest resistance was observed in beta-lactam antibiotics (49%), followed by aminoglycoside (30.2%) [33]. Additionally, Switzerland reported similar results, along with high resistance to tetracycline (6.9%), lincosamides (30.5%), and macrolides (11.1%) [113]. In Germany, clinical isolates exhibited high levels of resistance to gentamicin (85%), tetracycline (97.5%), fluoroquinolones (79%), and erythromycin (15.6%) [287]. Taken together, MRSA infections in horses are recognized as a broader threat within the equine community, particularly in veterinary hospitals. MDR staphylococci in equines are highly reported, and antimicrobial susceptibility testing is required before treatment decisions. Thus, proper antimicrobial stewardship and infection control practices are crucial in mitigating the spread of AMR in equines and preserving the effectiveness of antimicrobial therapies.

#### 7.1.6. *Listeria* Infections

*Listeria monocytogenes* is a Gram-positive motile rod-shaped bacterium that can be both aerobic and facultatively anaerobic, with the ability to survive intra-cellularly. This has been linked to diseases globally in humans, birds, and various livestock, such as cattle, llamas, sheep, and goats [296]. Listeriosis is frequently linked to foodborne gastrointestinal diseases that result in encephalitis, septicemia, and abortion in animals [296]. Though clinical disease in horses is exceedingly uncommon [297], it has been reported that infections in horses can cause multisystemic infections and abortions [298,299,300,301,302,303,304,305]. Intrauterine *Listeria* infections in mares resulted in the birth of a weak foal [306], with very low or no effect on the fertility of the mare [307]. In adult horses, *Listeria* has been recognized as a causative agent of meningoencephalitis and keratoconjunctivitis. However, instances of sepsis in adult horses or ponies have been reported very infrequently [303,306,308]. Similarly, *L. monocytogenes*-associated septicemia and diarrhea have been documented in newborn or older foals as well as adult ponies and horses [298]. *Listeria* transmission in horses commonly occurs through the fecal-oral route, where the brain is primarily affected, leading to encephalitis [309]. Entry into the nervous system typically happens through a wound in the oral mucosa [310]. On equine farms, improper fermentation of silage is frequently linked to cases of listeriosis, and contamination from groundwater or water tanks can also serve as sources of infection [311]. The treatment for listeriosis varies depending on the symptoms. For example, in the cases of kerato-conjunctivitis, commonly recommended medications include chloramphenicol, ampicillin, gentamicin, and a combination of oxytetracycline/polymyxin B [299]. However, in cases of neurological disorders, gentamicin is typically the preferred drug, although cefquinome has also been reported for use [312]. Some studies reported that the use of penicillin in combination with an aminoglycoside or other broad-spectrum antimicrobials is the best choice for listeria-associated hepatitis in foals [313]. Furthermore, broad-spectrum antibiotics, including amikacin, amoxicillin with clavulanate, and penicillin, can be used to treat abortion, pneumonia, and septicemia in horses [308].

*L. monocytogenes* has been exhibiting inherent resistance to several classes of antimicrobials, either because of the absence of antimicrobial targets (cefotaxime) or the presence of inherent resistance genes (fosfomycin, clindamycin, nalidixic acid, and sulfonamides) [314]. The acquired AMR in *Listeria* isolates from different species has been consistent worldwide, demonstrating resistance to cefotaxime, nalidixic acid, fosfomycin, sulfonamides, erythromycin, and clindamycin [315,316,317]. Few reports are available on the AMR of *L. monocytogenes* in horses. In Ohio, USA, *L. monocytogenes* isolated from three foals with septicemia demonstrated resistance to ceftiofur and lincomycin [308]. Similarly, Revold et al. reported that the *Listeria* isolates were found to be resistant to fucidic acid, which was isolated from a horse with keratoconjunctivitis [299]. *L. monocytogenes* poses a potential threat to equine health, although such cases are rare. While clinical cases are infrequent, if not treated in a timely manner, infections can lead to serious complications such as neurological diseases and septicemia in foals. The preventive measures should focus on controlling environmental sources of contamination, particularly improperly fermented silage, and ensuring clean water sources. Additionally, regular monitoring for AMR patterns and judicious use of antibiotics are essential for effective treatment and prevention of listeriosis in horses.

#### 7.1.7. *Enterococcus* Infections

*Enterococcus* spp. are gram-positive, facultative anaerobic bacteria that are present as a part of the normal gut microflora in horses [318]. While *Enterococcus* typically does not cause any symptoms in adult horses, it can lead to sepsis, lower urogenital tract infections, particularly umbilical infections, and watery diarrhea in foals [319]. *E. faecium* and *E. faecalis* are the primary species associated with these diseases in horses, though other species, such as *E. durans,* have also been detected [320]. In the USA, a surveillance study conducted from 1979 to 2010 reported the prevalence of Enterococci species in foals with sepsis to be 7.2% [321]. In Japan, a study conducted between 2015 and 2016 identified Enterococcus spp. in 97.2% of fecal samples from healthy foals, of which *E. faecium* was 54.1% and *E. faecalis* was 16.4% [322]. Similarly, in a case-control study conducted at the University of California, Davis, USA, between 2000 and 2017, isolated *Enterococcus* from various organs of the foals tested positive, including the lower urogenital tract (30%), respiratory system (20%), blood culture (10%), musculoskeletal system (12%), digestive system (19%), kidney (3%), and ophthalmological system (1%) [318]. The same study also reported a high case fatality rate of 52.1%, identifying *Enterococcus* as an emerging pathogen in foals [318]. Marsh et al. observed a 9.4% frequency of *Enterococcus* in blood cultures of foals admitted to the hospital between 1991 and 1998 [323]. Additionally, a study conducted in 2010 on a Thoroughbred breeding farm in central Kentucky, USA, demonstrated that 71% of foals with watery diarrhea tested positive for *E. durans* [320]. Similarly, a retrospective study conducted by Hollis et al. reported that 29.3% of foals suffering from diarrhea were found to be infected with *Enterococcus* between 1990 and 2007 [319]. In most cases, *Enterococcus* causes self-limiting diarrhea, for which supportive therapy is usually sufficient [324]. However, in more severe cases, patients suffering from sepsis require treatment using antibiotics such as cephalosporins, aminoglycosides, β-lactams, and/or potentiated sulfonamides, along with supportive therapy [318,321].

The treatment of enterococci-associated infections is typically challenging due to their intrinsic resistance to the aforementioned antibiotics [325]. However, the combination of penicillin and aminoglycosides has synergistic effects on this pathogen and is often recommended in foals [319]. Furthermore, acquired resistance to important antibiotics as a result of horizontal gene transfer has also been a major problem with this pathogen. Transferable *van* genes (*van*A and *van*B) along with *erm*(B) and *tet*(L) are responsible for resistance to vancomycin, macrolides, and tetracycline [326], respectively, and the prevalence of vancomycin-resistant *Enterococcus* (VRE) is due to the acquisition of these genes around 6.7–9.6% worldwide [327]. Similarly, in a 2023 study by Zowalaty et al., one of the two fecal samples collected from horses tested positive for *E. faecium* and showed resistance to oxacillin [326]. Marsh et al. reported VRE (8%) along with ampicillin (75%), sulfadiazine (67%), and Ticarcillin (75%) resistance [323]. In India, a study reported 80.2% VRE and 99% MDR isolates from sick horses [328]. A retrospective study conducted on foals with neonatal diarrhea reported that *Enterococcus* isolates demonstrated resistance to amoxicillin clavulanate (10%), cefoxitin (60%), ceftiofur (88%), chloramphenicol (26%), enrofloxacin (40%), imipenem (22%), and tetracycline (32%) [319]. Another study reported MDR *Enterococcus* isolates from foals with sepsis, which included fluroquinolones (28%), phenicols (13%), tetracycline (54%), and macrolides (41%) [318]. Although enterococci are opportunistic pathogens in horses, co-infections with other bacteria can further complicate treatment, especially under stressful conditions, and can cause life-threatening sepsis in foals [327]. The high-level resistance rates to antibiotics like vancomycin, tetracycline, and ampicillin highlight the urgent need for effective antimicrobial strategies. Therefore, accurate diagnosis and identification of their AMR patterns are crucial for effectively managing these infections. In order to combat MDR *Enterococci* in horses, it is necessary to implement stringent biosecurity measures, judicious use of antibiotics based on sensitivity tests, continued surveillance, veterinary guidance, and continuous education for staff and caretakers.

### 7.2. Bacterial Pathogens Affecting the Respiratory Tract of Horses

#### 7.2.1. *Rhodococcus equi* Infections

*R. equi* is a gram-positive, soil-borne, intracellular pathogen that primarily affects foals under six months of age [329]. While it predominantly impacts young foals, immunocompromised adult horses and humans are also vulnerable to this infection. Infected foals typically exhibit subacute to chronic purulent bronchopneumonia [46]. However, the bacteria are also known to cause extrapulmonary lesions such as mesenteric lymphadenitis, enterotyphlocolitis, abscess, and peritonitis in infected foals [330]. *R. equi* poses significant economic challenges to the equine industry [121,331]. *R. equi* is commonly found in soil [332]. Transmission of the bacteria typically happens when foals inhale soil particles contaminated with *R. equi* from heavily infected environments, such as farms where infected horses shed the bacterium through nasal discharge and feces [332,333,334]. Factors such as pathogen exposure [335,336,337,338,339,340,341], alteration in the animals’ innate and adaptive immune responses, and genetic factors all contribute to the transmission of infection to healthy foals [342,343,344]. The diagnosis of the infection is usually presumptive, and the exact morbidity is still unknown [345]. In regions where the infection occurs frequently, the treatments are prolonged and costly, leading to fatality rates potentially being substantial (8–80%) [346,347]. Despite high exposure and seroprevalence among foals, most of the infections are sub-clinical in nature, and only a subset of foals on endemic farms show clinical signs of the disease [348,349]. To detect sub-clinical infections and prevent severe *R. equi* pneumonia, endemic farms implement preventative measures such as routine thoracic ultrasounds and/or daily monitoring of body temperature for early detection of pneumonic lesions [350]. Once identified, the foals are treated with antimicrobials to prevent the onset of clinical signs [351]. The use of antibiotics in the treatment of sub-clinical cases of *R. equi* infections has highly contributed to the development of multidrug-resistant *R. equi* and the dissemination of the AMR genes into the environment [103]. The standard treatment for *R. equi* infections involves using various antibiotics. However, the treatment can be challenging due to the increasing drug-resistant strains and the lack of a clear protocol that specifies the best antimicrobial combination for animals infected with these strains [352]. Yet another issue is that *R. equi* is an intracellular pathogen, limiting the number of effective antimicrobials to only a few pharmacological classes [353]. Antibiotics like macrolides (erythromycin, azithromycin, and clarithromycin) have strong inhibitory efficacy against *R. equi* [354]. Combinations of rifampin with any of the macrolides can help in treating the infection. Alternatively, combinations such as gamithromycin, erythromycin, azithromycin, gentamicin, doxycycline, and SMZ with or without rifampicin can be employed [331,355,356,357,358,359].

Veterinarians commonly initiate antibiotic treatment for *R. equi* upon detection of pulmonary lesions during thoracic ultrasonography [360]. A study conducted in Kentucky, USA, in 2013 reported that out of the samples from 25 pretreated foals, 24% of the isolated *R. equi* demonstrated resistance to macrolides and rifampicin. Similarly, out of 13 foals post-treated with antibiotics, 62% of the *R. equi* isolates demonstrated resistance against rifampin and macrolides [139]. They also reported that 5% of isolates demonstrated resistance to azithromycin, clarithromycin, erythromycin, and rifampin in 2008, whereas in 2011 they reported 24% resistance to the same antibiotics [139]. Similarly, an increase of up to 40% in resistance against macrolides and rifampin in *R. equi* has been recorded [121]. In Kentucky, USA, between 1995 and 2017, the reported prevalence of resistance against both erythromycin and rifampin was 9.2% [352]. Another study in Kentucky, conducted between 2011 and 2017, reported that the prevalence of resistance to macrolides and rifampicin ranged between 15% and 24% [107,334]. Phylogenomic analysis has shown an increased prevalence of MDR-*R. equi*, attributed to the chromosomal *rpoB*^S531F^ mutation driven by prophylactic use of rifampin and macrolide therapies [361]. Though macrolides are the drug of choice for treating *R. equi* infections worldwide, some in vitro studies suggest that replacing macrolides and rifampicin with antimicrobials like doxycycline may offer the potential for reducing resistance [362,363,364].

*R. equi* represents a substantial health and economic challenge in the equine industry. Preventing *R. equi* infections is crucial for combating AMR and reducing losses associated with the disease [345]. While vaccination presents a promising alternative to prevent infections, most vaccine candidates tested so far have not proven to have sufficient effect [365]. Another avenue explored for prevention is the use of hyperimmune plasma, initially showing promise in protecting foals against both in vitro and natural infections [366,367]. Additionally, alternative treatments such as bacteriophages, gallium maltolate (GaM), and alternative antimicrobials have been investigated [368]. GaM has demonstrated effectiveness in inhibiting *R. equi* proliferation both extracellularly and within macrophages [360,369,370]. With current treatment strategies encountering rising AMR, there is a critical need to prioritize the judicious use of antibiotics. Moreover, research efforts and investments should prioritize the development of antibiotic alternatives and proactive surveillance of the bacteria, which are essential for improving both equine and public health outcomes.

#### 7.2.2. *Streptococcus equi* Subspecies *equi* Infections

*Streptococcus equi* ssp. *Equi* is a highly contagious bacterial pathogen that causes strangles in horses [371]. Foals up to the age of three months who are born to immune dams are usually immune due to maternal immunity [372]. Horses that are infected with *S. equi* exhibit prolonged clinical signs such as pyrexia, pharyngitis, and abscess development in the submandibular and retropharyngeal lymph nodes, which may obstruct or rupture into the upper respiratory tract [373]. Transmission of the bacteria occurs via direct contact with the discharges from the lymph nodes, nose, and eyes of sick and recovering horses [374]. Strangles can be zoonotic and transferred from sick animals to human beings [375]. The morbidity rate in susceptible horse populations is 85% to 100%, and mortality ranges from 4% to 8% [372,376]. The predisposing variables for disease transmission encompass overcrowding, the mixing of horses from different areas, and multiple stressors such as weaning, transportation, severe weather, concurrent sickness, and inadequate nutrition [377,378]. Most strangle cases resolve themselves without the use of antibiotics [372], and antimicrobial treatment is controversial [373,379]. Recent reports indicate that the majority of horses with *S. equi* infections will receive some form of antimicrobial therapy [380], although antibiotics can delay abscess maturation and potentially increase the risk of metastatic strangles [375].

Antibiotics like penicillin are commonly used to treat *S. equi* infections. Early initiation of antimicrobial treatment can enhance effectiveness, with procaine penicillin G being the recommended choice [381]. The bacterium is also susceptible to ampicillin, ceftiofur, rifampin, and SMZ [381]. However, in field conditions, antibiotics such as erythromycin, tetracycline, and beta-lactams have shown greater efficacy [382]. The rate of AMR development in *S. equi* is lower compared with other equine pathogens [383]. A study conducted in the UK discovered that *S. equi* isolates were sensitive to β-lactams (penicillin and ampicillin), SMZ, erythromycin, and cefoxitin but were resistant to tetracycline and streptomycin, whereas only one isolate was MDR [383]. Additionally, some sporadic reports of resistance have been observed worldwide, including macrolide-resistant isolates recovered in Germany [384], tetracycline resistance gene-containing isolates reported in Portugal [385], and β-lactam-resistant isolates reported in France, Japan, Belgium, and Portugal [385,386,387]. Though the incidence of *S. equi* resistance to most other drugs is low, the resistance to antimicrobials such as aminoglycosides such as gentamycin has been consistent. A study conducted in Kentucky, USA, demonstrated a 10.5% increase in gentamycin resistance in *S. equi* isolated between 2000 and 2010, and up to 91.3% resistance to sulfa drugs [388].

Despite being a significant and widespread cause of equine morbidity and mortality globally, there are few documented cases of successful control of natural outbreaks of *S. equi* infection (International Collating Centre Reports) [372,378,389]. The persistence of *S. equi* has led to its classification as an endemic disease in certain horse populations on specific premises. This persistence may be due to the survival of *S. equi* in carrier hosts [389,390,391] or in the environment [392]. Epidemiological studies have further suggested that the carrier state could be responsible for the ongoing presence of *S. equi* between epidemics [393,394]. Though, like any antibiotic, resistance can develop over time due to various factors, including overuse or improper use of antibiotics. Regular surveillance for antibiotic resistance in *S. equi* is also crucial for maintaining effective treatment options. 

### 7.3. Bacterial Pathogens Affecting Horses’ Reproduction

#### 7.3.1. *Streptococcus equi* Subspecies *zooepidemicus* Infections

*Streptococcus equi* ssp. *zooepidemicus (S. zooepidemicus)* is a β-hemolytic opportunistic commensal bacteria commonly found as a resident microflora of the caudal reproductive tract of horses. It is often associated with the development of equine endometrtitis, pyometra, ascending placentitis, and mastitis [395]. The bacteria can cause an ascending infection by traveling into the uterus, typically during natural mating or artificial insemination [396]. The vast majority of mares easily clear common bacterial contamination from the uterus during estrus, and the development of endometritis is dependent on mares’ physical uterine clearance mechanisms, the innate immune system of the host, and the presence of other stress factors such as iatrogenic conditions [88]. Treatment of endometritis generally relies on antibiotics, with penicillin as the first choice, along with uterine lavage and oxytocin if intrauterine fluid is present. Most equine *S. zooepidemicus* infections are susceptible to penicillin treatment [397,398], but a few studies have reported the development of AMR to penicillin in *S*. *zooepidemicus*, similar to *S. equi* [383]. While certain countries, such as the UK, have observed a temporal increase in AMR *S. zooepidemicus* strains, the trend has been inconsistent in the USA and Canada [363,397,399]. A recent study conducted in Kentucky in 2022 analyzed samples collected from 2012–2017 and found that out of 247 equine *S. zooepidemicus* isolates, approximately 99.6% of the isolates were resistant to at least one antibiotic tested, including cefazolin (6.9%), ceftazidime (2.5%), chloramphenicol (44.5%), enrofloxacin (96.2%), imipenem (1.6%), Azithromycin (4.1%), erythromycin (6.9%), penicillin (6.9%), oxacillin (3.8%), ticarcillin (2.5%), ticarcillin/clavulanate (3.4%), rifampin (5.5%), tetracycline (85.3%), doxycycline (33.3%), and Trimethoprim/sulfamethoxazole (30.3%). Moreover, more than half (53.3%) of the isolates were MDR, demonstrating resistance to phenicols, tetracyclines, fluoroquinolones, and potentiated sulfonamides [107]. Similarly, Awosile et al. reported that, between 1994 and 2013, up to 47% of resistance to tetracycline was recorded in Canada [397]. However, only 6% resistance to penicillin has been recorded in the UK, USA, and Canada [107,398,400]. The rising trend of AMR in *S. zooepidemicus* highlights the broader issue of AMR in the equine industry and its potential implications for both equine and human health. Therefore, concerted efforts are required from veterinarians, researchers, and policymakers to mitigate this threat and ensure sustainable health management practices in the equine industry.

#### 7.3.2. *Pseudomonas* Infections

The most common gram-negative pathogens affecting horses’ reproduction are *E. coli* and, to a lesser extent, *P. aeruginosa. E. coli* infections have been discussed under Section 7.1.2., and resistance patterns are similar for genital strains. *P. aeruginosa* is a gram-negative, opportunistic bacterium commonly found in the environment that frequently inhabits water [401,402]. Although *P. aeruginosa* is not a common pathogen in horses, it can cause opportunistic systemic infections in the reproductive tract, lower respiratory tract, eyes, skin, and guttural pouch. These infections are more likely to occur after antimicrobial therapy [402,403]. The severity of the infection can vary among mares. Infections caused by *P. aeruginosa* in the reproductive tract can result in endometritis as well as placentitis [404,405,406] and infertility [407,408]. While *P. aeruginosa* is classified as a venereal infection in horses [409], there is scant evidence to suggest that it is transmitted through sexual intercourse or artificial insemination [410,411], and the establishment of endometritis is highly dependent on mares’ uterine defense status [412]. Horse-to-horse transmission has been reported in some studies; however, several studies failed to demonstrate this route of transmission [409]. It is, however, plausible to consider that *P. aeruginosa* may be transmitted through contaminated surroundings [409]. Studies performed between the years of 1996 and 2021 in various countries such as the UAE, UK, USA, Italy, Egypt, Sweden, and Bahrain demonstrated that the prevalence of uterine infections of *P. aeruginosa* ranged between 0 and 11.7% [413]. Similarly, in 13 studies conducted between 2006 and 2021, LABÉO (Laboratoire d’Analyses Biologiques et d’Études de l’Ouest), Normandy, France, performed 35,686 antibiograms on equine samples, where *P. aeruginosa* was the fourth most prevalent pathogen, representing a prevalence of 3.6% [414]. However, *P. aeruginosa* strains detected in the respiratory tract (36.2%) and skin (17.1%) of horses were also reported in some studies [32,415].

The treatment of *P. aeruginosa* infections is particularly challenging due to the ability of the bacteria to exist as biofilms and their inherent resistance to many common antibiotics [90,416]. Additionally, it can acquire further resistance, leading to strains that are multi-resistant or extensively drug-resistant [416]. Antibiotics such as aminoglycosides are the primary drug of choice against *P. aeruginosa* in horses [417]. However, increasing AMR trends have been observed among *P. aeruginosa* isolates. For example, a study conducted by Bourély et al. in 2020 demonstrated resistant strains of *P. aeruginosa* to gentamycin (12.9%) and enrofloxacin (50.1%). These resistance strains were isolated from the reproductive tract of horses between 2012 and 2016 [31]. Similarly, another study conducted by Leon et al. in France between 2016 and 2019 reported resistant *P. aeruginosa* isolates. The findings showed resistance to cefquinome (11.9% in 2016, 14.3% in 2017, 14.7% in 2018, and 12.5% in 2019), gentamicin (10.2% in 2016, 8.6% in 2017, 14.7% in 2018, and 10.9% in 2019), and marbofloxacin (1.7% in 2016, 0% in 2017, 0% in 2018, and 4.7% in 2019) [415]. Furthermore, a study conducted by Keller et al. in the United States from 1993 to 2004 found an 11.8% prevalence of *P. aeruginosa* in cases of equine ulcerative keratitis. The same study reported complete resistance to ampicillin and chloramphenicol (100%), significant resistance to tetracycline and trimethoprim-sulfamethoxazole (83%), and resistance in half of the isolates to bacitracin and cephalothin (50%) [418]. In addition, *P. aeruginosa* isolated between 2016 and 2021 from endometrium or uterine lavage of mares in the UAE demonstrated resistance to amoxicillin-clavulanic acid (93%), amikacin (17%), chloramphenicol (83%), doxycycline (75%), enrofloxacin (8%), ceftiofur (91%), gentamycin (9%), oxytetracycline (83%), and SMZ (93%) [413]. Rising AMR in *P. aeruginosa* could pose a significant threat to the reproductive health of mares. Additionally, the formation of biofilms contributes to the complexities associated with *P. aeruginosa* infections in horses [419]. Proper prevention and control strategies are needed, along with regular AMR surveillance, to combat this pathogen in horses.

#### 7.3.3. *Taylorella equigenitalis*

*T. equigenitalis* infection, also known as “Contagious Equine Metritis” (CEM), is a major concern for the equine industry worldwide [420]. The infection primarily affects the reproductive tract of horses, resulting in economic losses due to disrupted breeding programs and decreased reproductive efficiency [421]. The horses suffering from CEM demonstrate vaginal discharge, endometritis, and infertility in mares, while stallions remain asymptomatic carriers [422,423]. The transmission of *T. equigenitalis* typically occurs through direct contact with infected genital secretions during natural mating or artificial insemination with contaminated semen, but can also occur through contaminated equipment during reproductive examinations [424]. The insidious nature of CEM, coupled with its potential for chronic carrier states in stallions, underscores the importance of stringent biosecurity measures and regular screening protocols to prevent its spread and minimize its impact on equine reproductive health and industry productivity [41,421,425,426,427].

*T. equigenitalis* isolates that were isolated in France in 1980 from cervical swabs of mares suffering from acute endometritis or cervicitis exhibited resistance to streptomycin (14.3%), clindamycin (100%), lincomycin (100%), and metronidazole (100%) [18]. In the UK, the same resistant pattern was observed among *T. equigenitalis* isolates with 100% resistance to streptomycin, clindamycin, lincomycin, and metronidazole [428]. The first outbreak of *T. equigenitali* in the USA occurred in Thoroughbred horses in Kentucky in 1978, following the importation of two infected carrier stallions from France in the fall of 1977 [429,430]. Economic losses stemming from this incident were conservatively estimated at $13.55 million [431]. Following this event, smaller outbreaks occurred among Trakehners in Missouri in 1979 [432], Thoroughbreds in Kentucky in 1982 [433], and sub-clinical cases in imported Lipizzaners in Wisconsin in 2006 [434]. From 2000 to 2012, the International Collating Centre of the Animal Health Trust documented 146 confirmed cases of CEM across 12 countries, primarily affecting non-Thoroughbred horses. Other outbreaks reported in the USA (2008–2009), South Africa (2011), the United Kingdom (2012), and Denmark (2021) exclusively involved non-Thoroughbred and artificial-breeding populations. Over a 12-year span, 74% of confirmed *T. equigenitalis* carriers were stallions, while 26% were mares [424,426,427,435,436].

The treatment of *T. equigenitalis* infections in horses mainly relies on the use of antibiotics, including macrolides, such as erythromycin and azithromycin, as well as aminoglycosides like gentamicin and spectinomycin [437]. These antibiotics are often administered via systemic or intrauterine routes [438]. In cases of chronic carriers, local antibiotic treatment coupled with antiseptics may be necessary to achieve clearance of the organism. Overall, a comprehensive treatment approach involving antibiotic therapy, biosecurity protocols, and monitoring for recurrence is crucial for effectively managing *T. equigenitalis* infections in horses [424]. Despite the development of effective antibiotic treatments and biosecurity measures, the control of this disease is complicated by the existence of asymptomatic carriers and the emergence of AMR strains. The historical and ongoing outbreaks highlight the critical need for robust screening protocols, vigilant surveillance, and adherence to treatment guidelines to manage the spread of this infectious disease and mitigate its economic repercussions on equine breeding operations. The USA has been implementing specific restrictions and rigorous testing regulations for the importation of horses from countries with CEM to keep the disease out of the domestic horse population.

#### 7.3.4. *Klebsiella* Infections

*Klebsiella pneumoniae* is considered a commensal organism, residing as a normal microflora in the digestive and urogenital systems of horses [64]. *K. pneumoniae* is a common cause of nosocomial pneumonia in horses; however, the bacteria is also responsible for causing endometritis and infertility in mares [439,440]. Another common manifestation of *K. pneumoniae* is diarrhea in foals [441]. *Klebsiella*-associated pneumonia is a common complication of mechanical ventilation in horses [442]. However, the transmission of bacteria causing diarrhea occurs through the consumption of contaminated food and water [439]. Similarly, uterine infections in mares are usually due to venereal transmission by the infected stallion [411]. This bacterium is normally found in the soil and water [443]. The presence of virulence factors such as O-lipopolysaccharide, adherence factors, capsular antigens, and siderophores enables this bacterium to survive in challenging environments [444]. A study conducted in Japan demonstrated that the *K. pneumoniae* K1 strain was found to be one of the major causes of equine contagious metritis in mares from 1980 to 1986. They also reported that 89.8% of the *K. pneumoniae* isolates were of the K1 capsular type [440]. Another study in Japan isolated 27 *K. pneumoniae* strains from diarrheal foals on a metritis-infected farm during 1982–1983 [441]. In the USA, a study reported a prevalence of 7.9% of *K. pneumoniae* in mares suffering from metritis during 2015–2016 [445]. Another study conducted from 2010 to 2017 in Italy showed that 34% of the uterine swabs tested positive for *K. pneumoniae* [446]. *K. pneumoniae* infections are commonly treated with ceftiofur, gentamicin, SMZ, penicillin, metronidazole, enrofloxacin, doxycycline, chloramphenicol, and rifampin [64,442]. However, the rise in AMR among this bacterium has become a significant concern in both medical and veterinary fields [447,448].

Health agencies worldwide have classified MDR *K. pneumoniae* producing extended-spectrum β-lactamases (ESBL) as an urgent threat [449]. A study conducted in Japan reported 3.3% ESBL-producing *K. pneumoniae* isolates from healthy Thoroughbred racing horses, and all of them were found to be MDR, with 100% of the samples resistant to ampicillin, cefotaxime, cefuroxime, tetracycline, oxytetracycline, and doxycycline, followed by ceftazidime (83.3%), gentamicin (75.0%), kanamycin (66.7%), streptomycin (8.3%), and chloramphenicol (8.3%) [450]. Similarly, Rathbone et al. found that 31.8% of the bacteria isolated from endometrial swabs of breeding mares in the U.K., collected between 2014 and 2020, were tested positive for *K. pneumoniae*. These isolated bacteria were found to be resistant to penicillin (97.2%), ampicillin (97.1%), nitrofurazone (87.5%), SMZ (58.3%), polymyxin (41.7%), oxytetracycline (19.4%), neomycin (16.7%), and gentamicin (5.6%) [451]. In France, 2.4% of *K. pneumoniae* isolates were found in horses, with 43% of these isolates originating from reproductive systems. The presence of MDR isolates was reported to range from 17% to 39.7% (with a rather stable average of around 21%) during the period of 2006–2016 [32]. Köhne et al. documented that 3.9% of *K. pneumoniae* isolates were found in endometrial swabs of mares in Germany from 2018 to 2022. The isolates exhibited resistance to the following antibiotics: SMZ (15.33%), penicillin (100%), gentamicin (1.39%), marbofloxacin (1.05%), enrofloxacin (1.74%), cefquinome (4.88%), ceftiofur (5.23%), tetracycline (8.01%), and amoxicillin (98.95%) [452]. Similarly, Ferrer et. al. demonstrated that out of 45 uterine samples from mares suffering from metritis in the USA between 2006 and 2016, 6.9% of the samples were positive for *Klebsiella* spp. They also demonstrated that 90% of the *Klebsiella* isolates were MDR with resistance to penicillin (100%), erythromycin (100%), rifampin (100%), cefazolin (55.6%), gentamycin (50%), tetracycline (50%), and SMZ (40%) [445]. Furthermore, a study conducted at North Carolina State University, USA, found *K. pneumoniae* isolates in 18% of the horses suffering from pneumonia. The same study demonstrated that the *K. pneumoniae* isolated from those adult pneumonic horses was resistant to amikacin (10%), chloramphenicol (30%), doxycycline (30%), enrofloxacin (43%), gentamycin (35%), tetracycline (39%), and SMZ (45%) [439]. Although *K. pneumoniae* is usually a harmless commensal organism in horses, MDR strains represent a significant risk to equine health. The situation is further complicated by the potential for zoonotic transmission to humans, which can result in severe infections. In horses, *K. pneumoniae* should be considered in the differential diagnosis of conditions such as metritis and pneumonia, and antibiotic sensitivity testing is strongly recommended before initiating treatment. Given the seriousness of the infections caused by MDR strains, prompt and precise diagnosis along with suitable antimicrobial therapy are essential for effective management and mitigation of these threats.

#### 7.3.5. *Amycolatopsis* spp. and *Crossiella equi* Causing Nocardioform Placentitis

Focal mucoid placentitis, also known as nocardioform placentitis (NP), is a distinct form of equine placentitis that is associated with focal mucoid placenta inflammation and is limited to the chorionic surface of the ventral aspect of the placenta without reaching the fetus [93]. The disease was first diagnosed in central Kentucky (KY) in 1986 [453]. Since then, Kentucky has experienced outbreaks in 1998, 1999, 2011, 2017, and 2020 [454,455]. Sporadic cases have also been reported in Florida [456], Louisiana [457], South Africa [458], Italy [459], Australia [460], and most recently in New Zealand [461]. NP is caused by gram-positive, branching bacteria called actinomycetes. These include *Amycolatopsis* spp. and *Crossiella equi*, with more recent identifications of *Streptomyces atriruber* and *Streptomyces silaceus* strains playing a role [93,462]. The singular nature and specific localization of NP lesions are inconsistent with an ascending or hematogenous bacterial etiology [93]. While some common soil bacteria (actinomycetes) are believed to be the cause, attempts to find specific types (*Amycolatopsis* spp. and *Crossiella equi*) in the environment of affected mares in Kentucky were unsuccessful [463]. Experimental induction of the disease in mares via intrauterine inoculation of *Crossiella equi* at breeding or through various routes in pregnant mares was unsuccessful [92]. Consequently, the pathogenesis of the disease remains obscure. A study of the 2011 Kentucky outbreak revealed that *Amycolatopsis* spp. (49% of cases) was the most common isolate, followed by *Crossiella equi* (29% of cases) [457]. The treatment of the clinical cases of the disease is conducted using antibiotics such as SMZ, penicillin, and tetracycline [454,464].

A recent report investigated the antimicrobial susceptibility of *Amycolatopsis* spp. and *Crossiella equi* isolated from clinical NP cases during the 2019–2020 foaling season in Kentucky and found that more than 50% of the *Amycolatopsis* spp. isolates (*n* = 91) were resistant to tobramycin (51.7%), imipenem (59.3%), and cefepime (61.5%) [93]. The same report found that more than 80% of the *Crossiella equi* isolates (*n* = 60) were resistant to clarithromycin (83.3%), cefepime (83.4%), imipenem (85%), tobramycin (90%), amikacin (90%), and ciprofloxacin (91.7%) [93]. Similarly, previous in vitro studies indicated the susceptibility of *Nocardia* spp. to SMZ, leading to its widespread use for nocardioform placentitis treatment [465]. However, a recent report identified resistance to SMZ in 19.8% of *Amycolatopsis* spp. and 5% of *Crossiella equi* isolates [93]. This emergence of AMR in *Nocardia* spp. underscores the growing concern about AMR within the equine reproductive tract. Together, AMR is a growing concern in equine reproduction. For instance, antibiotics are commonly used in semen extenders to prevent bacterial growth during transport, but this practice raises concerns about promoting AMR. This highlights the need for more judicious use of antibiotics and the exploration of alternative strategies for preventing bacterial contamination in equine reproduction.

### 7.4. Bacterial Pathogens Affecting Musckloskeletal/Joints of Horses

Musculoskeletal disorders pose a significant challenge in the equine industry, contributing to wastage illnesses in Thoroughbred horses [466] and other breeds [467], thus resulting in substantial economic losses [468]. Several bacterial infections can impair the musculoskeletal system in horses [469]. Although bacterial infections may not primarily cause musculoskeletal diseases, consequences from other preceding events can make the condition worse [470]. For example, septic arthritis and septic tenosynovitis are common in horses, with clinical signs including lameness, edema, effusion, local hyperthermia, and pain sensitivity [471]. Similarly, osteomyelitis can be a consequence of the internal fixation of open and closed fractures [470]. Bacterial infections can occur in bone, tendon, joint sheath, or bursa and can cause bone destruction [472]. The most frequently isolated bacterial group causing musculoskeletal disorders includes Enterobacteriaceae, streptococci (non-β-hemolytic or β-hemolytic), staphylococci (coagulase-positive or negative), and *Clostridium* spp. [473]. These organisms are often found to form biofilms on bone surfaces or implants, exhibiting their pathogenic effects. Typically, they cause complications in the wound healing process [474]. Horses can also suffer from deadly infectious diseases caused by the Clostridial genus, including botulism, tetanus, and muscular abscesses [475], caused by toxins produced by *C. botulinum* and *C. tetani*, respectively, which are rarely diagnosed in horses worldwide [476,477,478].

Antibiotic treatments are consistently recommended for musculoskeletal infections to avoid complications during the healing process. Thus, a combination of cephalosporin and amikacin, which cover a broad range of effectiveness against various bacteria, including Enterobacteriaceae, *Streptococcus*, and *Staphylococcus* [479]. Previously, it was reported that chloramphenicol, amoxicillin-clavulanic acid, and gentamycin were mostly effective against *Staph. aureus*; however, they were not effective against coagulase-negative staphylococci, streptococci, and *Actinobacillus* [480]. Similarly, chloramphenicol, rifampin, and SMZ have also been used for musculoskeletal infections [481,482]. These antibiotics should not be used to prevent diseases due to the risk of the development of AMR [483].

AMR has been observed in organisms isolated from the musculoskeletal system. A study conducted in Ohio, USA, from 1979 to 1989 revealed high resistance rates among enterobacteriaceae isolates that cause septic arthritis/tenosynovitis or osteomyelitis, notably against SMZ (72.9%), kanamycin (65%), cephalothin (57%), chloramphenicol (45%), amoxicillin-clavulanic acid (35%), and gentamicin (33%). *Pseudomonas* isolates also showed complete resistance (100%) to amoxicillin-clavulanic acid, cephalothin, and chloramphenicol, with similar resistance patterns observed in coagulase-positive *Staphylococcus* isolates [470]. In another study conducted in Brazil, synovial fluid from horses with arthritis revealed bacterial pathogens in 45 out of 60 samples. The identified pathogens included *S. equi* subsp. *equi* (18.3%), *E. coli* (15.0%), *Staph. aureus* (10.0%), *S. equi* subsp. *zooepidemicus* (8.3%), *S. intermedius* (3.3%), *Proteus vulgaris* (3.3%), *Trueperella pyogenes* (3.3%), *P. aeruginosa* (3.3%), *K. pneumoniae* (1.7%), *R. equi* (1.7%), *Staph. epidermidis* (1.7%), *K. oxytoca* (1.7%), *Nocardia asteroides* (1.7%), and *Enterobacter cloacae* (1.7%). The AMR profile showed significant resistance rates to penicillin (42.2%), enrofloxacin (33.3%), and amikacin (31.2%) [473,484]. The formation of biofilms in the musculoskeletal system, especially on the bones or transplant surface, exacerbates wound healing complications, prolongs treatment durations, and increases costs. Moreover, the escalating AMR among these pathogens underscores the critical need for enhanced surveillance, management strategies, and advanced research aimed at developing novel antimicrobial agents or alternative therapeutic approaches.

### 7.5. Bacterial Pathogens Affecting the Neurological Health of Horses

Neurological diseases are relatively uncommon in horses; however, they can lead to substantial economic losses within the equine industry. Infectious conditions like bacterial meningitis and brain abscesses are commonly documented neurological conditions affecting horses globally [485,486,487]. These conditions can result from bacterial infections in the meninges or subarachnoid space, which may occur through direct transmission or hematogenous spread, posing a fatal risk to the infected horses [488,489]. *Staph. aureus*, *Streptococcus* spp., and gram-negative enteric bacteria such as *E. coli* are the most commonly identified pathogens in septic foals with bacterial meningitis [485]. For example, histological examination of the brain of a 3-year-old Quarter Horse displaying neurological signs revealed the presence of *S. pluranimalium* in active lesion sites, which was confirmed through PCR [490]. Similarly, extraintestinal pathogenic *E. coli* (ExPEC) has the ability to invade the intestinal mucosa, enter the blood stream, and eventually cross the blood-brain barrier [491]. These organisms can also invade the central nervous system (CNS) through direct inoculation, including penetrating wounds or surgical wounds [492]. In horses, the development of meningitis is often associated with sepsis [493]. A study conducted by Viu et al. in Spain from 2004 to 2009 diagnosed 10 septic foals with meningoencephalitis and detected *E. coli* from both blood culture and CSF [494]. However, in adult horses, other bacteria such as Cryptococcus neoformans [495], *S. equi* subsp. *equi* [496], *S. equi* subsp. *zooepidemicus* [497], *S. suis*, *Actinomyces* spp., *K. pneumoniae*, *E. coli*, *Actinobacillus equuli*, and *Pasteurella caballi* have also been isolated [493,498].

Antimicrobials are often used to treat different infectious neurological conditions; however, many antimicrobials cannot effectively cross the blood-brain barrier to combat bacterial pathogens. This limitation restricts the selection of effective antimicrobial therapies [499]. However, lipophilic drugs such as chloramphenicol, rifampin, and fluoroquinolones can swiftly diffuse across tight junctions via the transcellular pathway, enabling them to effectively interact with the pathogens [499]. Antibiotic resistance is another limitation in treating such infections. In a study conducted among five horses that died after developing meningitis after sinus surgery in five different European clinics between 2005 and 2010, one horse was found to have been infected with MRSA, and another had ESBL *E. coli*, which was isolated from the sinus [500]. Furthermore, Fu et al. (2021) isolated macrolide- and lincosamide-resistant *S. pluranimalium* from a 3-year-old Quarter Horse with meningoencephalitis [490]. Another study from Spain has indicated that *E. coli* strains isolated from cases of meningoencephalitis exhibit considerable antibiotic resistance. Specifically, resistance was noted to ceftriaxone in 20% of cases, cephalexin and cefotaxime each in 33.3% of cases, and amoxicillin-clavulanic acid in 40% of cases. Additionally, half of the isolates were resistant to tetracycline, enrofloxacin, cefoperazone, and trimethoprim-sulfamethoxazole [494]. Hence, the emerging resistance to common treatments, such as macrolides and lincosamides, underscores the urgent need for ongoing research and development of more effective therapeutic strategies to manage these critical infections effectively and preserve the health and economic viability of the equine population.

### 7.6. Bacterial Pathogens Affecting the Urinary Tract of Horses

Urinary tract infections (UTIs) in horses typically arise from compromised host defenses, including obstruction of urinary flow due to masses or strictures, uroepithelial damage from trauma, or alterations in the normal bacterial flora of the urethra, vulva, or prepuce [501]. The most frequently isolated organisms from horses with cystitis are *E. coli*, *Enterobacter*, *Klebsiella*, *Corynebacterium*, *Proteus*, *Streptococcus*, *Staphylococcus*, and *Pseudomonas*, although all except *Klebsiella* and *Corynebacterium* can also be found in cultures of normal equine urine [502]. Additionally, *Streptococcus equi*, *Actinobacillus equuli*, and *R. equi* have been identified as urinary pathogens in foals with sepsis, potentially contributing to septic nephritis of hematogenous origin [503]. Similarly, other diseases such as equine leptospirosis are extensively documented, with a recent report from Japan detailing cases of nonulcerative kerato-uveitis in affected horses [504]. The primary therapeutic approach for horses with UTIs involves administering antimicrobials like penicillin, cephalosporins, and sulfonamides to eradicate the causative organisms [505]. Furthermore, ampicillin and combinations of penicillin with an aminoglycoside, ceftiofur, trimethoprim-sulfonamides, or enrofloxacin are also viable treatment options [506]. However, multiple studies have reported the emergence of AMR among bacterial strains isolated from equine urinary tracts. The Equine Hospital at the University of Zurich found that 45% of *E. coli* and 18% of *Enterococcus* isolates exhibited MDR resistance to penicillin, cephalosporin, macrolides, and tetracycline [507]. Additionally, ESBL *E. coli* has been detected in equine urine samples in the Netherlands [508]. In Sweden, extensive AMR in the *Enterobacter cloacae* complex from equine urinary tract infections has been reported, including resistance to beta-lactam antibiotics such as ampicillin, penicillin, amoxicillin with clavulanic acid, combination, and cephalosporine, along with aminoglycosides like gentamicin and streptomycin, and trimethoprim-sulfamethoxazole [509]. Due to insufficient data, the status of AMR in equine urinary tract infections is not well addressed. However, based on the reports reviewed, it is inferred that AMR is on the rise and the presence of MDR pathogens could pose a serious risk to the equine industry. Understanding and addressing AMR in equine urinary tract infections is crucial for safeguarding equine health, promoting responsible antimicrobial use, and preserving treatment efficacy for the future.

## 8. Conclusions

Antimicrobials will remain indispensable for managing bacterial infections in horses. Many of these infections are zoonotic or zooanthroponotic. The widespread use of antibiotics in animal production systems as growth promoters, therapeutic agents, and disease preventative agents against bacterial infections are some of the most important driving factors that aid in the development of MDR strains. AMR is a complex phenomenon, and controlling the spread of MDR pathogens within the equine industry demands a multifaceted approach that can address such complexity. Effective strategies must prioritize the judicious use of antimicrobials in human and veterinary medicine and agricultural practices. This review highlighted the importance of proper use of antibiotics in equines, the epidemiology of equine infectious diseases, and worldwide AMR reports. The absence of sufficient AMR surveillance reports was one of the major limitations of this review. Unlike the poultry, swine, and cattle industries, the equine industry lacks comprehensive regulatory frameworks on this topic. Following the guidelines provided by national advisory committees such as the Presidential Advisory Council on Combating Antibiotic-Resistant Bacteria (PACCARB), which recommends comprehensive action plans to address AMR globally, can be instrumental in the fight against AMR. Recently, in 2024, PACCARB published a report on AMR in which PACCARB focused on the four major fundamental concepts of infection prevention, awareness, development, and access to combat AMR. It places emphasis on the following strategies: (1) prioritizing infection prevention, evidence-based diagnostics, proper sanitation, hygiene, wastewater management, agricultural biosecurity, and infection control in healthcare and veterinary settings, (2) increase awareness among the general public and political leaders regarding the public health threat, the economic impact, and potential social issues driven by AMR to stimulate national and global actions, (3) develop incentives to promote the identification and development of alternative therapies, including new diagnostics and therapeutics, to address AMR, and (4) ensure equitable access to essential and non-essential antimicrobials, vaccines, and diagnostics based on local and regional needs, while promoting stewardship and the optimal use of these resources. In addition to these recommendations, improving surveillance systems to monitor the prevalence and dissemination of resistant bacterial strains is essential for the early detection and management of AMR, which is vital in combating the spread of disease. New pathogen detection techniques, including whole genome sequencing and AMR detection techniques, can be introduced to increase the accuracy and frequency of surveillance. This requires the establishment of robust laboratory networks capable of promptly and accurately identifying and controlling MDR pathogens. Moreover, implementing stringent regulations and guidelines for prescribing and dispensing clinically essential antimicrobials is integral to combating antibiotic misuse and alleviating the selection pressure driving resistance. As recommended by PACCARB, educating healthcare professionals, veterinarians, farmers, and the public about the risks associated with the misuse of antimicrobial agents is crucial from a One Health perspective. This educational initiative is essential for fostering antimicrobial stewardship and promoting behavioral changes among all stakeholders involved in the equine industry. By increasing awareness of the consequences of antimicrobial misuse and the importance of responsible antimicrobial use, we can empower individuals to make informed decisions and contribute towards the collective efforts currently being made to combat AMR. In summary, addressing AMR in the equine industry demands a multifaceted approach, emphasizing prudent antimicrobial use, robust surveillance, regulatory measures, improved biosecurity, and innovative research. By adopting these strategies collectively, we can mitigate the threat of AMR and safeguard the health and wellbeing of equine, animals, and humans alike.

## Figures and Tables

**Table 1:** Antimicrobials that are being used to treat equines infections/diseases and their reports of antimicrobial resistance worldwide.

Antimicrobial Class	Example Drugs	Bacteria	Disease in Horse	Side Effects	Use in Other Hosts	AMR Reports
Penicillin	Benzyl Penicillin/Penicillin G	*S. equi* sub. *zooepidemicus*; *Staphylococcus*, *Actinobacillus*, *Clostridium perfringens*, *Corynebacterium pseudotuberculosis*, *Listeria monocytogenes*, *Dermatophilus congolensis*	Bacterial pneumonia, Lung abscess, Guttural Pouch Empyema, Pleuropneumonia, Strangles, Clostridial myositis, Pigeon fever, Stromal abscess, Cystitis, Pyelonephritis, Endometritis, Placentitis, Mastitis, Dermatophilosis	Excitement, seizure-like activity, muscle soreness, focal myositis, respiratory difficulty, diarrhea, head shaking, teeth grinding, salivation, lacrimation, high borborygmus, mild colic, passage of soft feces, and colitis.	Birds, Cattle, Camels, Goats, Rabbits, Sheep, Pigs, Fish, Human	- *S. equi* sub. *Zooepidemicus*: USA (10%) [107], Italy (34.8%) [108], England (2.5%) [109], South Africa (37.4%) [110], France (2.2%) [31]. - *Staphylococcus*: USA (78.6%) [111], Spain (37.8%) [112], Switzerland (76.9%) [113], Nigeria (53.5%) [114], South Africa (63.7%) [110], France (42.6%) [31]. - *Actinobacillus*: England (16%) [109], UK (100%) [115], South Africa (80.5%) [110].
Aminopenicillin	Ampicillin, Amoxicillin	*Salmonella*, *E. coli*, *Proteus*, *Pseudomonas*, *Klebsiella*, *Enterobacter*, *Streptococcus*, *Staphylococcus*	Salmonellosis, Cystitis, Pyelonephritis, Endometritis	Irritation and antimicrobial-associated colitis [116].	Birds, Cattle, Camels, Goats, Rabbits, Sheep, Pigs, Fish, Human	- *Salmonella*: USA (21.6%) [49], Thailand (16.67%) [117], South Africa (24.1%) [110]. - *Pseudomonas*: England (7.7%) [109], South Africa (95.1%) [110]. - *E. coli*: USA (86%) [118], UK (45.6%) [119], England (35.4%) [109], South Korea (5.2%) [34], South Africa (78.4%) [110], France (4.5%) [31]. - *Proteus*: South Africa (92.3%) [110]. - *Klebsiella*: South Africa (98.9%) [110]. - *Enterobacter*: South Africa (95.2%) [110]. - *Streptococcus*: South Africa (55.6%) [110]. - *Staphylococcus*: South Africa (67.3%) [110].
Aminopenicillins in combination with beta-lactamase inhibitors	Amoxicillin-clavulanate	*Streptococcus* spp., *Pasteurella* spp., *Actinobacillus* spp., *Escherichia coli*, *Klebsiella* spp., *Pseudomonas aeruginosa*, *Clostridium* spp.	Bacterial Pneumonia, Pleuropneumonia, Neonatal Sepsis, Joint Infections, Urinary Tract Infections, Skin and Soft Tissue Infections, Endometritis, Enterocolitis		Birds, Cattle, Camels, Goats, Rabbits, Sheep, Pigs, Fish, Human	- *Streptococcus* spp.: England (0.7%) [109]. - *Actinobacillus* spp.: England (0.6%) [109]. - *E. coli*: USA (25%) [118], UK (8.8%) [119], England (8.7%) [109], South Korea (2.1%) [34]. - *Klebsiella* spp.: France (28%) [120] - *P. aeruginosa*: England (7.7%) [109].
3rd and 4th generation cephalosporins	Ceftiofur, Cefovecin, Cefquinome	*Staphylococcus*, *E. coli*	Cellulitis, Folliculitis, furunculosis, Endometritis	May cause discomfort, irritation, diarrhea, and colitis.	Cattle, Goats, Sheep, Pigs, Rabbits, Birds, Human	- *Staphylococcus:* USA (3.7%) [33], South Africa (30.1%) [110]. - *E. coli:* USA (23%) [118], England (14%) [109], South Africa (44%) [110], France (7.6%) [31].
Aminoglycosides	Gentamicin, Streptomycin, Neomycin, Amikacin	*Bordetella bronchiseptica*, *Streptococcus*, *Salmonella*, *Staphylococcus*, *E. coli*, *Actinobacillus*, *R. equi*, *Brucella abortus*, *Taylorella equigenitalis*	Guttural Pouch Empyema, Salmonellosis, Septic arthritis, Fistulous withers, Septic tenosynovitis, Endometritis, Placentitis, Contagious equine metritis (CEM), Seminal vesiculitis	Nephrotoxicity, ototoxicity, and muscle irritation reported in horse	Birds, Cattle, Goats, Rabbits, Sheep, Pigs, Bees, Fish, Camel, Human	- *Streptococcus:* South Africa (87.4%) [110], France (8.3%) [31]. - *Staphylococcus:* USA (30.2%) [33], England (24.9%) [109], South Africa (23.9%) [110], France (22.1%) [31]. - *Salmonella:* Thailand (16.67%) [117]. South Africa (12.1%) [110]. - *E. coli:* USA (63%) [118], England (23.4%) [109], South Africa (29.5%) [110], UK (9.3%) [119], South Korea (1%) [34], France (9%) [31]. - *Actinobacillus:* England (32.2%) [109], South Africa (43.9%) [110].
Amphenicols	Chloramphenicol Florfenicol	*Streptococci*, *R. equi*, *E. coli*	Liver abscess, Brain abscess	Anemia and pancytopenia	Birds, Cattle, Goats, Rabbits, Sheep, Fish, Pigs, Horses	- *Streptococci:* England (13.7%) [109], South Africa (7.3%) [110]. - *R. equi:* South Africa (71.4%) [110]. - *E. coli:* USA (34%) [118], England (26.5%) [109], South Africa (21.2%) [110].
Fluroquinolones	Enrofloxacin, Marbofloxacin	*Staph. aureus*, *Streptococcus*, *Actinobacillus*	Folliculitis and furunculosis, Otitis interna-media	Noninflammatory arthropathy, weakening and rupture of tendons, ataxia, severe oral ulceration, colitis, and neurologic behaviors.	Dogs, Cats, Poultry	- *Staph. aureus:* USA (4%) [33], England (13.1%) [109], South Africa (21.2%) [110], France (68.4%) [31]. - *Streptococcus:* South Africa (46.2%) [110], England (27.9%) [109]. - *Actinobacillus:* England (3.7%) [109], South Africa (12.2%) [110].
Macrolides	Erythromycin, Tylosin, Azithromycin	*R. equi*, *Streptococcus* spp.	Rhodococcal Pneumonia, Chronic Respiratory Disease	Colitis, diarrhea, fever, and hepatobiliary toxicity.	Bee, Birds, Cattle, Goats, Rabbits, Sheep, Fish, Pigs, Horses	- *Streptococcus* spp.: England (15.4%) [109], France (8.3%) [31]. - *R. equi:* USA (0.6%) [121].
Sulphonamides and pontentiated sulphonamides	Sulfamethoxazole + trimethoprim	*Streptococcus*, *R. equi*, *Corynebacterium pseudotuberculosis*, *Salmonella*, *E. coli*, *Proteus*, *Pseudomonas*, *Klebsiella*, *Enterobacter*, *Staphylococcus*, *Actinobacillus*	Abdominal abscess, Salmonellosis, Stromal abscess, Pyelonephritis, Cystitis, Brain abscess, Spinal abscess, Otitis interna-media, Liver abscess, Nocardioform placentitis, Placentitis, Vaginitis, Folliculitis and furunculosis, Pastern dermatitis, Staphylococcal pyoderma	Dysbiosis, colitis and diarrhea occasionally, tremor, excitement, ataxia, collapse, dysrhythmia, and hypotension.	Dogs, Cats, Horses, Cattle, Poultry	- *Staphylococcus:* USA (24.4%) [33], England (34.6%) [109], South Africa (31.4%) [110]. - *Actinobacillus:* South Africa (12.2%) [110]. - *E. coli:* USA (66%) [118], UK (55.9%) [119], South Africa (51.3%) [110], South Korea (9.4%) [34], France (26.2%) [31]. - *Klebsiella*: South Africa (54.8%) [110], France (15.5%) [31]. - *R. equi*: South Africa (71.4%) [110], USA (30.3%) [122]. - *Salmonella*: South Africa (27.6%) [110]. - *Pseudomonas*: South Africa (72%) [110]. - *Proteus*: South Africa (53.8%) [110]. - *Enterobacter*: South Africa (38.1%) [110]. - *Streptococcus*: France (9.9%) [31], South Africa (14.7%) [110].
Tetracyclines	Doxycycline, oxytetracycline	*S. equi* sub. *Zooepidemicus*, *Staphylococcus*, *Actinobacillus*, *E. coli*	Pneumonia, Proliferative enteropathy, Wound	Renal tubular necrosis, hypotension, and antimicrobial-associated colitis.	Birds, Cattle, Goats, Rabbits, Sheep, Pigs, Fish, Camel, Bees, Horses	- *S. equi* sub. *Zooepidemicus*: England (33.8%) [109], South Africa (24.1%) [110]. - *Staphylococcus*: USA (26.8%) [33], England (35.6%) [109], South Africa (40.7%) [110], France (60.1%) [31]. - *Actinobacillus*: England (5.8%) [109], South Africa (12.2%) [110]. - *E. coli*: USA (81%) [118], England (48%) [109], UK (50.7%) [119], South Africa (80.5%) [110], South Korea (9.4%) [34], France (23.1%) [31].
Nitroimidazoles	Metronidazole	*C. difficile*, *C. perfringens*, *C.tetani*	*Clostridium difficile* associated diarrhoea, *Clostridium perfringens*-associated diarrhoea, Proximal enteritis, Tetanus	Depression, weakness, ataxia, vestibular signs, seizures, peripheral neuropathy, and anorexia.	Dogs, Cats	- *C. Difficile:* USA (19.04%) [123]
